# Personalized mRNA vaccines in glioblastoma therapy: from rational design to clinical trials

**DOI:** 10.1186/s12951-024-02882-x

**Published:** 2024-10-04

**Authors:** Iman Karimi-Sani, Zahra Molavi, Samaneh Naderi, Seyedeh-Habibeh Mirmajidi, Iman Zare, Yasaman Naeimzadeh, Atena Mansouri, Amir Tajbakhsh, Amir Savardashtaki, Amirhossein Sahebkar

**Affiliations:** 1 Department of Medical Biotechnology, School of Advanced Medical Sciences and Technologies, Shiraz University of Medical Sciences, Shiraz, Iran; 2 Proteomics Research Center, Shahid Beheshti University of Medical Sciences, Tehran, Iran; 3 Research and Development Department, Sina Medical Biochemistry Technologies Co. Ltd., Shiraz, 7178795844 Iran; 4 Department of Molecular Medicine, School of Advanced Medical Sciences and Technologies, Shiraz University of Medical Sciences, Shiraz, Iran; 5 Cellular and Molecular Research Center, Birjand University of Medical Sciences, Birjand, Iran; 6 Pharmaceutical Sciences Research Center, Shiraz University of Medical Sciences, Shiraz, Iran; 7 Infertility Research Center, Shiraz University of Medical Sciences, Shiraz, Iran; 8 Biotechnology Research Center, Pharmaceutical Technology Institute, Mashhad University of Medical Sciences, Mashhad, Iran; 9 Applied Biomedical Research Center, Mashhad University of Medical Sciences, Mashhad, Iran; 10 Department of Biotechnology, School of Pharmacy, Mashhad University of Medical Sciences, Mashhad, Iran

**Keywords:** Personalized medicine, mRNA vaccine, Glioblastoma, Clinical trials, Brain cancer, Brain tumor

## Abstract

Glioblastomas (GBMs) are the most common and aggressive malignant brain tumors, presenting significant challenges for treatment due to their invasive nature and localization in critical brain regions. Standard treatment includes surgical resection followed by radiation and adjuvant chemotherapy with temozolomide (TMZ). Recent advances in immunotherapy, including the use of mRNA vaccines, offer promising alternatives. This review focuses on the emerging use of mRNA vaccines for GBM treatment. We summarize recent advancements, evaluate current obstacles, and discuss notable successes in this field. Our analysis highlights that while mRNA vaccines have shown potential, their use in GBM treatment is still experimental. Ongoing research and clinical trials are essential to fully understand their therapeutic potential. Future developments in mRNA vaccine technology and insights into GBM-specific immune responses may lead to more targeted and effective treatments. Despite the promise, further research is crucial to validate and optimize the effectiveness of mRNA vaccines in combating GBM.

**Graphical Abstract:**

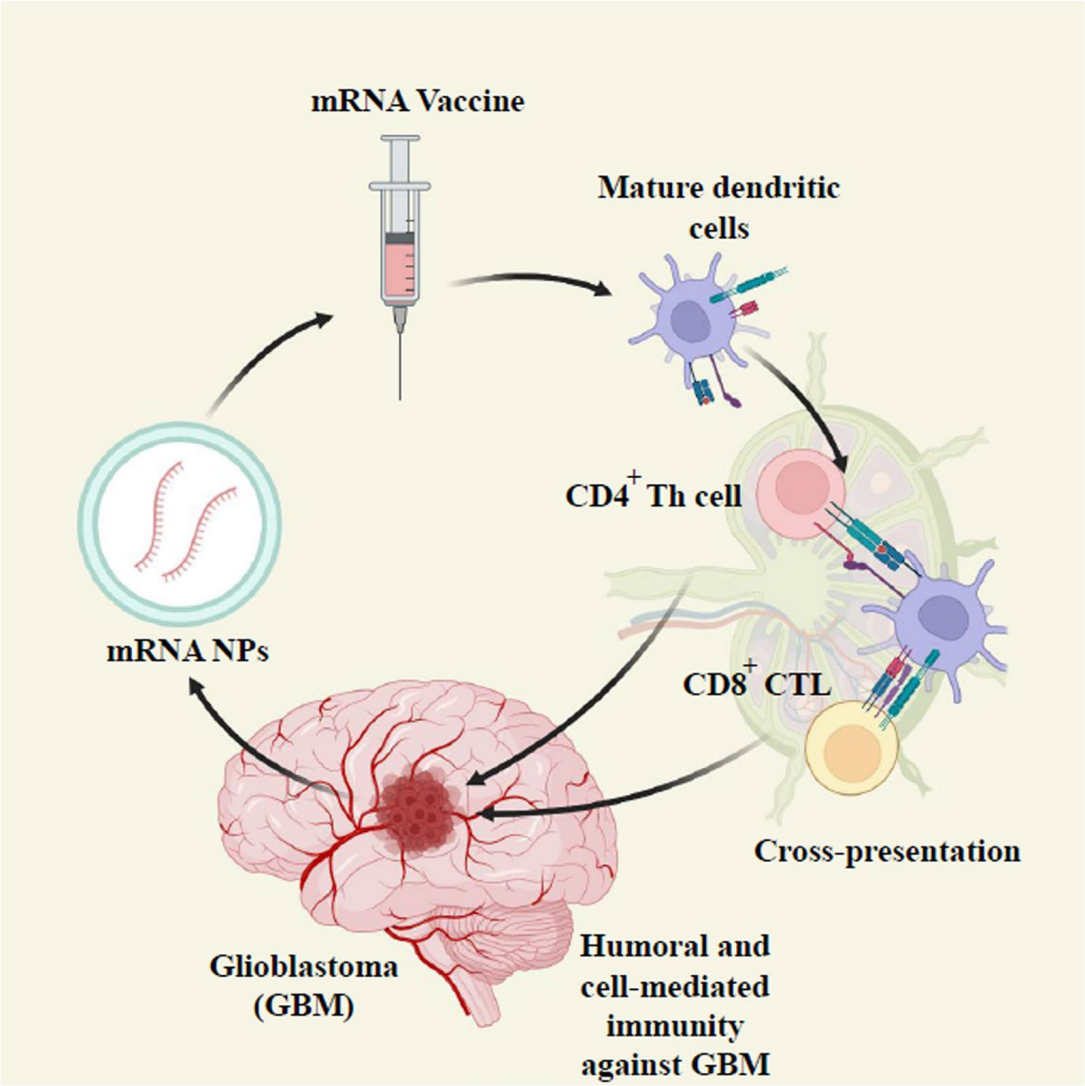

## Introduction

Glioblastomas (GBMs), the most common and aggressive human brain tumors, account for nearly half of all malignant primary brain tumors [1]. Brain tumors share some characteristics and challenges with tumors elsewhere in the body, but they also present special problems due to the unique properties of their organs [2]. As the most frequent brain tumor, GBMs are classified according to the World Health Organization (WHO) classification system, but some tumor types rarely become malignant, such as meningiomas and neurinomas [3]. The prevalence of this cancer increases with age, peaking at 15.2 cases per 100,000 in individuals aged 75–84 years, despite a low annual incidence compared with other cancer types. In patients older than 75 years, survival rates decline with age, with only 3.3% surviving 2 years after diagnosis [4]. Notably, approximately one-third of children and adolescents diagnosed with GBM survive for 2 years. The prognosis remains gloomy, particularly for elderly patients, where median survival is less than 4 months with the best supportive care alone [1, 5]. Since advanced age, poor performance, and incomplete resection are established negative prognostic factors, more effective treatment strategies are urgently needed.

The human brain, with its intricate micro-anatomy, is the most complex organ in the body, comprising a diverse array of cell types. While most brain cells transition into a post-mitotic state during adulthood, a select group known as neural stem and progenitor cells (NSPCs) retain the replication capacity, potentially contributing to learning, memory, and tissue repair post-injury. NSPC, found in areas, such as the sub-ventricular zones, sub-cortical white matter, and hippocampi of the temporal lobes in adults, have been implicated as the probable source of GBMs [1]. Notably, most adult GBM originates in brain regions housing NSPCs, predominantly in the temporal (19.7%), parietal (12.2%), and frontal (25.8%) lobes. While GBMs primarily manifest in the brain, they can also emerge in the spinal cord (4.3%), brainstem (4.2%), cerebellum (2.9%), and occipital lobe (3.2%) [5].

The comprehensive management of newly diagnosed GBM requires a multidisciplinary approach. In the current treatment protocols, surgical resection in combination with concurrent radiation and adjuvant chemotherapy using the alkylating agent temozolomide (TMZ) is the gold standard treatment. The intricacies of GBM, characterized by frequent invasiveness and localization in critical brain areas, including those governing speech, motor function, and sensory perception, pose challenges for extensive and complete surgical resection. Radical removal of the primary tumor mass, hindered by invasiveness, proves non-curative, leaving infiltrating tumor cells in the surrounding brain and paving the way for disease progression or recurrence. Studies have demonstrated improved outcomes with a larger extent of surgical resection, indicating the need for aggressive resection when possible. Statistically significant associations between increased resection extent and prolonged progression-free survival (PFS) and overall survival (OS) have been consistently observed in various studies. By integrating functional magnetic resonance imaging (MRI), diffusion tensor imaging (DTI), ultrasound, computed tomography (CT) scans, and MRI with direct stimulation during surgery, advancements in surgical techniques and preoperative mapping allow multimodal neuro-navigation while integrating anatomical and functional data specific to each patient. Despite these technological advances, distinguishing between normal brain tissue and residual tumor remains a substantial challenge [1, 6, 7]. Moreover, brain tumors frequently demonstrate diversity, as distinct cell populations exhibit differing responses to treatments [8]. As a result, personalized treatment approaches adapted to individual patients are imperative.

Immunotherapy is a promising avenue in brain cancer treatment, leveraging the body’s innate and adaptive defenses to target and eliminate malignant cells. Immune checkpoint inhibitors, such as pembrolizumab and nivolumab have demonstrated efficacy in specific brain cancer types by obstructing proteins that hinder immune responses, thereby empowering immune cells to identify and combat malignant cells. The use of novel immunotherapeutics, including monoclonal antibodies, tumor antigen-based vaccines, and chimeric antigen receptor (CAR) T cells, is under evaluation. Current immunotherapy methods aim to enhance immune function against tumor cells, utilizing diverse approaches such as blocking PD-1, PD-L1, and CTLA4, using cancer vaccines, or administering engineered immune cells, such as natural killer (NK) cells or CAR T cells. These therapies target immunological dysfunction to prompt the host’s immune system response, aiming for a specific immune reaction against tumor cells. Immune checkpoint blockade, targeting CTLA4, PD-1, and PD-L1 which was previously effective in cancers, such as melanoma, holds promise for recurrent GBM. These diverse approaches boost the host’s immune system response, potentially improving survival and quality of life for patients with GBM [9, 10].

Immunotherapy approaches directly address cancer biomarkers, induce tumor regression, and modify the inhibitory tumor microenvironment (TME), all of which are geared toward enhancing the OS rates. However, challenges persist in the effectiveness of immunotherapy for brain cancer. The blood–brain barrier (BBB) limits immune cell entry to the tumor site, potentially compromising treatment outcomes. Moreover, the suppressive brain microenvironment and tumor diversity add complexity to the activating immune responses. Myeloid-derived suppressor cells (MDSCs) and tumor-associated macrophages (TAMs), diverse myeloid cells found in GBM tissues and blood, hinder immune responses. While MDSCs suppress T-cells, TAMs play pro-tumor roles. The complexity of the TME limits the immune cell functionality, and dysfunctional NK cells are common. The absence or dysfunction of tumor-infiltrating lymphocytes (TILs) hampers the response to immunotherapy, emphasizing the significance of immune checkpoints, such as PD-1/PD-L1 interactions and the need for therapies that consider the complexity of the TME and its role in immunosuppression within GBMs [8, 11–14]. Understanding these dynamics is crucial for developing effective GBM-specific immunotherapies. A comprehensive understanding of the intricate interplay between the immune system and brain tumor cells is imperative to forge more potent and tailored treatment strategies. Because cancer vaccines train the immune system to recognize and eliminate tumor cells, this form of immunotherapy has received considerable attention.

The field of mRNA vaccines is rapidly evolving, marked by accumulating substantial preclinical data and the initiation of numerous human clinical trials in recent years. In this review, we delve into the existing approaches of mRNA vaccines for GBM, consolidating recent discoveries, highlighting obstacles and notable successes, and providing insights into the future trajectory of mRNA vaccines. Evidence suggests that mRNA vaccines hold promise for overcoming several hurdles encountered in vaccine development for infectious diseases and cancer.

## mRNA-based cancer vaccines

In general, four types of cancer vaccines are available: vaccines based on tumor cells/immunity cells, vaccines based on peptides, vaccines based on nucleic acids, and vaccines based on viral vectors. Nucleic acid-based vaccines, such as mRNA vaccines, have become a popular area of research due to their unique advantages, including specificity, safety, and ease of manufacturing [15–17]. Among these, mRNA vaccines exhibit intriguing traits akin to DNA, and their research and development are garnering increasing attention. Unfortunately, their adoption remains widespread and accepted within the medical community.

### mRNA-based dendritic cell vaccines

**Fig. 1:**
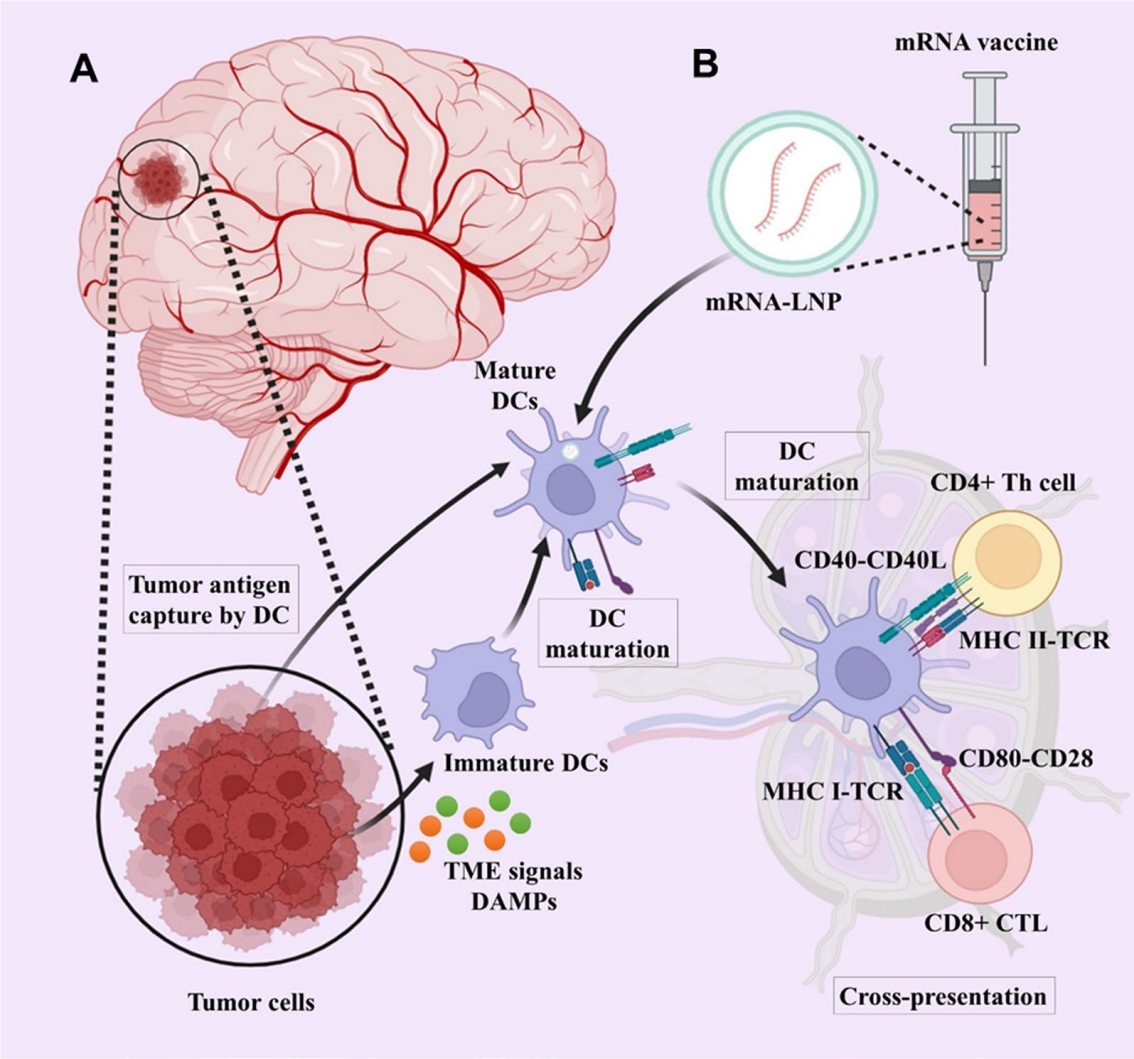
The presentation of tumor antigens by DCs to T cells. The concept of mRNA cancer vaccination involves delivering mRNA that encodes tumor antigens into immune cells and producing translated proteins. Transformed antigens can be displayed on immune cells to be recognized by the immune system, which will then produce antibodies against tumor cells. DCs are activated by damage-associated molecular patterns (DAMPs) from tumor cells in the tumor microenvironment. **A** A mature DC captures tumor antigens or **B** expresses mRNA vaccine antigens and migrates to tumor draining lymph nodes to cross-present tumor antigens presented on MHC I molecules. Mature DCs migrate into CD4^+^ T and CD8^+^ cell areas. Finally, effective antitumor responses are induced with cross-presentation by activating and cross-priming CTLs. Created with BioRender.com

The patient’s immune system’s ability to differentiate between healthy and tumor cells, relying on the presence of antigens in the tumor, is a fundamental aspect of active specific immunotherapy [18]. Within this category, the most hopeful and harmless approach to cancer treatment is therapeutic cancer therapy, specifically involving the utilization of dendritic cells (DCs)-based strategies [19, 20]. This treatment uses the patient’s DCs, which exhibit the antigen characteristics of tumor cells. DCs, which are specialized antigen-presenting cells (APCs), have a crucial role in connecting the intrinsic and adaptive immune reactions. An immune response will occur if DCs are transfected mRNA of an expected tumor antigen and delivered to the host (Fig. 1).

### mRNA-based direct cancer vaccines

A substitute for DC vaccines involves using mRNA directly, eliminating the need for DC separation, cell culture, and subsequent re-administration. Indigenous cells, including APCs, internalize the introduced mRNA and transport it to the cytoplasm for translational processing.

### mRNA-encoded antibodies

To expedite clinical advancement, mRNA molecules are additionally being used for the conveyance of complete IgG antibodies or frameworks, encompassing bispecific engineered antibodies while tackling pharmacokinetic and production complications. Monoclonal antibodies (mAbs) are recognized as an established approach for combating cancer, aiming at tumor cells and modifying the immune system reactivity.

#### mRNA-encoded antigen receptors

Harnessing T cells to target tumors presents considerable potential in the cancer treatment landscape. The redirection process operates through the durable integration of antigen receptors specific to tumors. These receptors can be either T cell receptors (TCRs), which identify MHC-presented epitopes from intracellular and extracellular antigens, or CARs, which adhere to the surface antigens of tumors independently of MHC involvement.

### mRNA-encoded immunomodulators

In recent studies, mRNA-based immunomodulators have encompassed various components, including cytokines, costimulatory ligands, and receptors. Achieving a substantial protein yield is a key objective when manufacturing immunomodulators from cellular messenger RNA.

## Delivery systems and administration routes for mRNA cancer vaccine

**Fig. 2:**
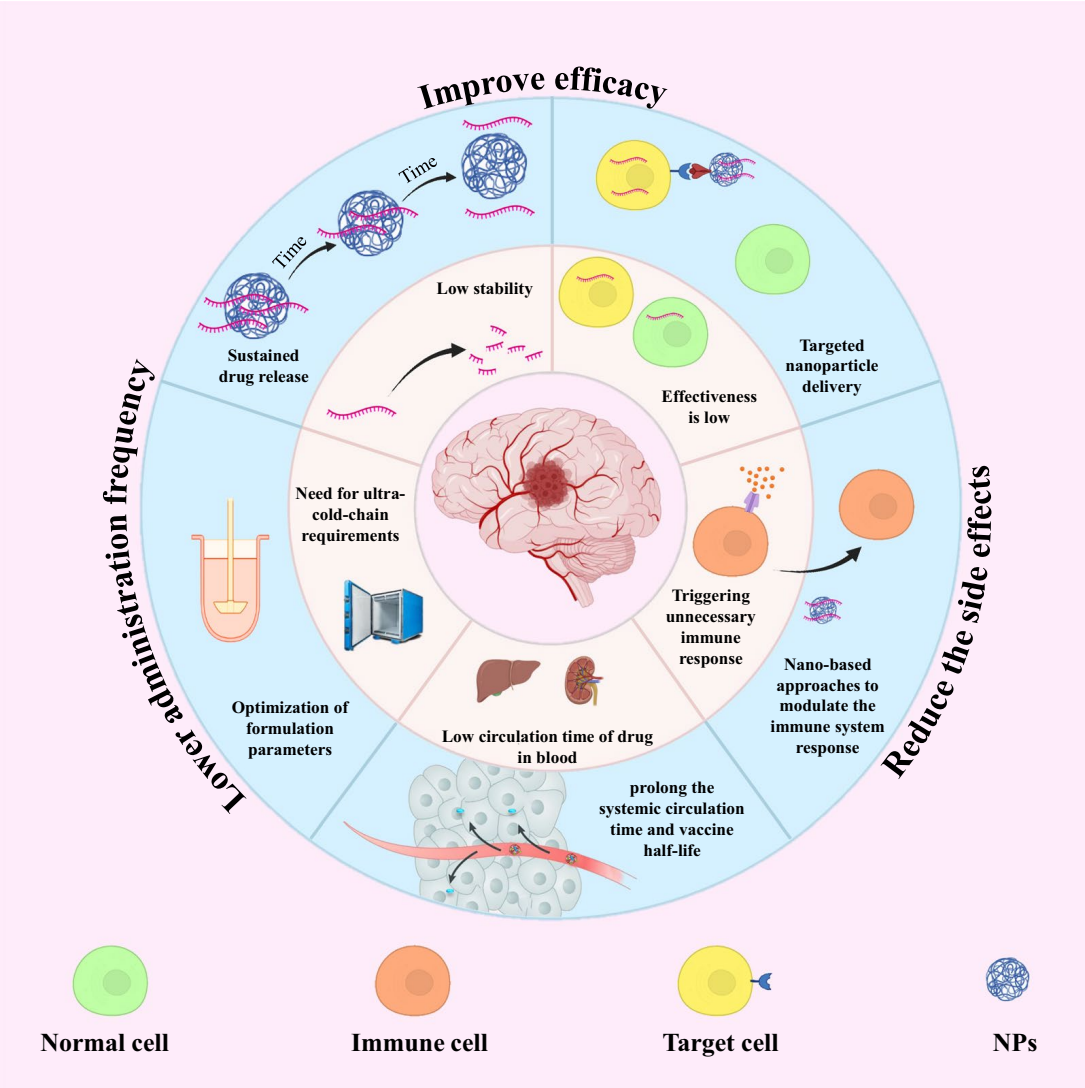
Advantages of nano-based mRNA vaccine delivery systems. Created with BioRender.com

The choice of the route of administration and the efficient delivery system into the cytoplasm of target cells are also important factors to obtain the desired therapeutic effect. Nevertheless, the effective transportation of mRNA molecules into cells poses additional obstacles as they must overcome tissue, extracellular, and intracellular barriers on their way to the targeted site. After that, mRNAs can be quickly eliminated by the immune system, broken down by nuclease enzymes in the extracellular environments, repelled by the plasma membrane, captured by endosomes, and broken down through internal defense mechanisms [21]. Hence, the main challenge currently faced is the unstable nature of mRNA molecules and their susceptibility to degradation by nucleases. Moreover, mRNA must stay structurally intact and reach the desired tissue/cells at a sufficient concentration in order to be translated into effective proteins for treating brain disorders [22, 23]. Researchers have explored numerous approaches for delivery of mRNA vaccines. Although, mRNA vaccines have been delivered in the form of free mRNA, nanocarriers like lipid-derived and polymer-derived materials have greatly enhanced the uptake of mRNAs by cells, leading to significant interest in recent times (Fig. 2).

### Naked mRNA

Unlike carrier-based mRNA vaccines, naked mRNAs are administered through direct injection of the mRNA solution. While naked mRNAs do not easily penetrate cell membranes, various studies have put forth hypotheses regarding their uptake mechanisms. Some researchers propose that the uptake of naked mRNA occurs via DC-mediated macropinocytosis. This process facilitates the expression of the antigen-encoding mRNA and enhances the activation of T cells and DCs. Once DCs reach maturity, they degrade the mRNAs [24, 25]. The commonly utilized solutions for naked mRNA are Ringer’s solution and lactated Ringer’s solution [26, 27]. Both solutions are enriched with calcium, which enhances the uptake of mRNA [28]. Research has been conducted on the intranodal and intradermal administration of naked mRNA. In a study by Sebastian et al*.*, a significant immune response was observed following the intranodal injection of naked mRNA containing a single epitope in murine models [29]. In addition, Sonia and colleagues explored the intradermal delivery of naked mRNA that encodes a fluorescent protein into excised pig skin, demonstrating that this method resulted in protein expression [30]. The researchers discovered that administering naked mRNA intradermally led to the expression of proteins. While these studies did not address certain aspects such as clinical applications, mRNA dosages, and levels of expression, they provide evidence of concept and the feasibility of using naked mRNA. Among the various administration routes, intradermal delivery is favored over intranasal delivery due to the latter’s complexity, whereas the dermis is abundant in APCs [31]. Nonetheless, naked mRNA, as an exogenous nucleic acid, is readily identified by the immune system and is quickly degraded by nucleases once it enters the body [32].

### Nanosystems for mRNA delivery

**Fig. 3:**
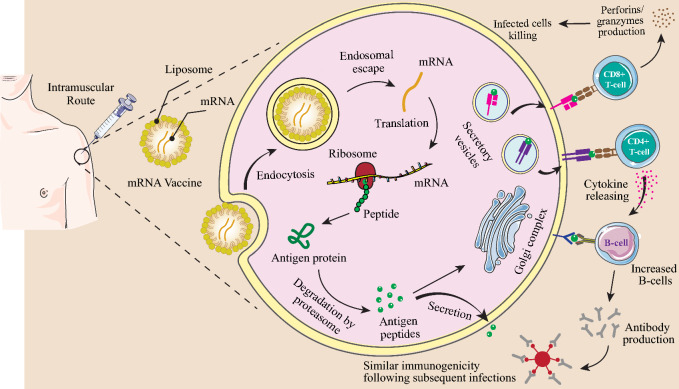
An overview of mRNA vaccine immunogenicity. DCs, macrophages, and B cells present antigens to the immune system through mRNA vaccines. The tumor antigen is secreted out of the host cell or converted into smaller peptides by the proteasome after translation termination. An antigenic peptide is processed in the endoplasmic reticulum and the Golgi apparatus, and then loaded on MHC class I or class II. DCs uptake soluble tumor antigens and stimulate CD4^+^ and CD8^+^ T cells with antigen-specificity by using MHC-II and cross-presentation pathways. In addition to activating antigen-specific CD8^+^ T cells in DCs through the MHC-I pathway, endogenously expressed tumor antigens can also induce humoral and cell-mediated immune responses. This aggressive germinal center response follows mRNA vaccination, which leads to B-cells identifying tumor antigens and producing antibodies that can neutralize tumor cells. Created with BioRender.com

A variety of nanovehicles, including lipid-based nanoparticles (NPs), polymeric NPs, and lipid-polymer hybrid NPs, has garnered significant interest in the delivery of mRNA [33–35] (Table 1). Non-viral nanocarriers offer several benefits: (a) they can effectively condense mRNA, protecting it from enzymatic degradation [36]; (b) they enable efficient targeting and delivery of mRNA to lymphatic organs, such as lymph nodes and APCs, which enhances antigen uptake and presentation, thereby increasing vaccine efficacy; (c) these nano-delivery systems facilitate endosome escape following endocytosis, thereby enhancing transfection efficiency [34] (Fig. 3).

**Table 1:** Types of nanosystems to deliver mRNA vaccine

	Types of nanosystems	Advantages	Challenges	Ref.
	Naked mRNA	• Easy to store and prepare • Easy to scale up	• Prone to RNase degradation • Low delivery efficiency	[32, 157, 158]
	Lipid nanoparticles	• Protect mRNA from RNase degradation • Reduced toxicity • Efficient intracellular delivery of mRNA • Tissue tropism • High reproducibility • Easy to scale up	• Potential side effects • Less drug entrapment • Serious issue with sterilization • Chemically unstable	[159–161]
	Polymer nanoparticles	• Protect mRNA from RNase degradation • Higher stability • Various methods of preparation • Efficient intracellular delivery of mRNA	• Potential side effects • Difficult scalability • Insufficient toxicity analysis • Polydispersity	[162, 163]
	Peptide-based nanoparticles	• Protect mRNA from RNase degradation • Protamine-mRNA complex has adjuvant activity	• Low delivery efficiency • mRNA complexed with protamine is translated poorly	[61, 164, 165]
	Virus-like replicon particle	• Protect mRNA from RNase degradation • Produced by cell free systems • Efficient intracellular delivery of self-amplifying mRNA • Presence of disulfide bond provides stability • Strong expression	• Challenging to scale up • Less stable • Escape phagocytosis • Extravasate from blood vessels • Antibody production against viral vectors	[166–168]
	Cationic nanoemulsion	• Protect mRNA from RNase degradation • Squalene-based CNEs have adjuvant activity • Formulation can be prepared and stored without RNA for future use • Easy to scale up	• Limited delivery efficiency	[131, 169, 170]

### Lipid nanoparticles for mRNA delivery

Lipid NPs (LNPs) have become a prominent focus within the biopharmaceutical industry, recognized for their potential as delivery systems for a range of nucleic acid therapies, including mRNAs. These NPs offer numerous benefits, such as low immunogenicity, substantial payload capacity, ease of production, and excellent scalability. In the context of mRNA delivery, LNPs present significant advantages, including straightforward formulation, modular design, biocompatibility, and a high capacity for mRNA payloads. Acting as intelligent, nanoscale lipid carriers, LNPs facilitate the transport of mRNA into the cytosol. These nanocarriers can efficiently deliver mRNA into cells by fusing with the lipid bilayer of early endosomes, thereby releasing the mRNA into the cytoplasm while protecting it from RNase degradation during systemic circulation [37]. Typically, LNPs are composed of three primary components: an ionizable lipid (40–50%), cholesterol (38–45%), and a helper phospholipid (10–12%), with the occasional addition of a PEGylated lipid (1–2%) [38]. These components work synergistically to encapsulate and protect the mRNA. The first lipids utilized for RNA delivery were the cationic DOTMA (1,2-Di-*O*-octadecenyl-3-trimethylammonium propane) and its synthetic counterpart DOTAP (1,2-Dioleoyl-3-trimethylammonium-propane), introduced in 1989 [39]. Since then, a variety of other cationic lipids, including the well-known Lipofectamine, have been utilized for RNA delivery [40]. Notably, mRNA-LNP vaccines for COVID-19 are currently in clinical use, representing a groundbreaking approach to mRNA-based therapies [41].

LNPs have emerged as highly effective nano-delivery systems for the targeted administration of nucleic acid therapies in the treatment of GBM. A notable example is a Phase I clinical trial (NCT06389591) investigating RNA-lipid particle (RNA-LP) vaccines for recurrent adult GBM, which aims to assess the feasibility of manufacturing, safety, and the maximum tolerated dose (MTD) in adult patients with recurrent GBM. This clinical trial represents the first human Phase I study focused on RNA-LP vaccines for recurrent adult GBM. In addition, another Phase I trial (NCT04573140) is exploring RNA-LP vaccines for newly diagnosed adult patients with unmethylated MGMT GBM and pediatric patients with newly diagnosed high-grade glioma (pHGG). This study is structured into three phases: Surgery, Radiation, and Immunotherapy. The RNA-LP vaccination will commence within 4 weeks post-radiation, following an evaluation of the post-radiation MRI for baseline assessment. After radiation therapy, participants will receive three RNA-LP vaccines at 2-week intervals, leading to 12 cycles of monthly adjuvant RNA-LP vaccines, culminating in a total of 15 vaccinations. Participants may continue to receive RNA-LP vaccines for a duration of up to 14 months.

Various types of NP platforms are available for the delivery of the GBM vaccine. Researchers have sought to incorporate a range of distinctive molecules, including X-hydroxycholesterol [42], PEG-lipid [43–45], iBL0713 (an ionizable lipid) [46], N-series lipidoids [47], synthetic ionizable lipidoids [48], DOTAP [49], etc*.*) into LNP formulations. This integration aims to enhance targeted delivery, improve transfection efficiency, and increase the rate of endosomal escape. The findings from these investigations indicate that LNPs optimized for composition possess significant potential to address the challenges associated with mRNA delivery [50].

### Peptide-based nanoparticles for mRNA delivery

A variety of peptides serve as carriers for the delivery of mRNA vaccines. When utilized as the main carrier for RNA delivery, peptides should possess a positive charge. Cationic peptides, characterized by a high content of arginine and lysine residues, provide positively charged amino groups that facilitate the complexation with nucleic acids through electrostatic interactions [51, 52]. Among these, protamine is the sole peptide carrier that has been assessed in clinical trials for mRNA vaccines [53]. Protamine, a cationic peptide, has been employed in numerous early investigations concerning mRNA vaccine delivery. In aqueous solution, protamine and mRNA spontaneously form a complex, with the size of this complex being influenced by the concentration of NaCl [54]. Additionally, the protamine–mRNA complex exhibits significant adjuvant activity, demonstrating immunogenic properties due to its structural resemblance to viral RNA genomes [55, 56].

The viability of the mRNA–protamine complex was evaluated using β-galactosidase–mRNA–protamine, which was administered into a GBM tumor. Results indicated that the mRNA complexed with protamine exhibited poor translation efficiency [57, 58]. Furthermore, protamine was utilized in conjunction with CureVac’s self-adjuvanted RNActive® delivery technology to form complexes with mRNA, contributing to the development of vaccines for rabies and influenza A [59, 60]. It is noteworthy that protamine has been shown to enhance the transfection efficiency of complexed nucleic acids without any detectable cytotoxicity at concentrations up to 10 mM, in contrast to other commonly used transfection agents, such as polyethylenimine (PEI) polymer, which has been reported to induce in vitro toxicity at doses exceeding 5 mM [61]. Recently, the application of protamine in mRNA delivery has gained traction for the formulation of established vaccines, and further research is anticipated. However, as of now, this nanocarrier has not been employed in clinical studies for GBM vaccines.

### Polymer nanoparticles for mRNA delivery

Polymeric NPs have garnered significant attention in recent years for extensive biomedical applications, establishing themselves as a fundamental component of the nanobiotechnology field. Both natural and synthetic polymers serve as adaptable materials, providing numerous benefits such as biodegradability, biocompatibility, and non-toxicity. The encapsulation of therapeutic agents within polymeric NPs facilitates sustained drug release, thereby prolonging their half-life. This characteristic is advantageous for enhancing drug efficacy and safety, minimizing adverse side effects, and improving patient acceptance and adherence [62, 63]. Additionally, hydrogels represent another promising application within nanotechnology, particularly for the delivery of immunotherapeutics. A hydrogel consists of a crosslinked hydrophilic polymer capable of suspending organic substances, including proteins and nucleic acids. Although RNA-loaded hydrogels are still under development for GBM, preliminary studies have shown their effectiveness in vitro against triple-negative breast cancer [64]. These hydrogels can be utilized as scaffolds to embed and deliver therapeutics, potentially countering the rapid evolution and heterogeneity associated with GBM [65]. Various nanotechnology-based delivery systems, including lipoplexes, polyplexes, and lipid-polymer hybrid NPs, have also gained considerable interest and have been investigated for mRNA delivery [34].

## Synthesis of mRNA in vitro and sequence engineering of synthetic mRNA

Messenger RNA (mRNA), known as a substitute genetic material for the expression of the protein, has garnered significant interest. Regarding expressing target proteins, mRNA has several advantages compared with conventional plasmid DNA, highlighting the inherent distinctions in mRNA molecules. mRNA directly changes into the protein through a single translation step in the cytoplasm. This allows the attainment of high protein levels, akin to viral systems. It also ensures swift onset times and controlled expression of target proteins, tailoring the pharmacodynamic effects as needed. Nonetheless, the practical utilization of mRNA has encountered certain limitations stemming from various factors. These include the inherent instability of mRNA, potential immunological hurdles, and, notably, the absence of efficient methodologies for mRNA synthesis [66–68]. In the 1990s, these constraints were overcome through the advancement of in vitro transcription (IVT) techniques for mRNA synthesis.

mRNA is a novel method of vaccination that involves the expression of protein antigens in pathogens or tumor cells. This activates the host’s immune system, thereby eliminating tumor cells and preventing infections [69, 70]. The utilization of conventional mRNA has faced previous challenges, including low stability and heightened immunogenicity. Consequently, the application of conventional mRNAs in clinical trials was constrained. Nonetheless, Kariko et al*.* demonstrated that substituting cytidine and uridine with 5-methylcytidine and pseudouridine in mRNA molecules rendered them more resilient in biological fluids [71]. This alteration significantly mitigated the immune system activation [71–74], paving the way for the clinical application of modified mRNA. To induce protein expression, the selected gene transcript may be delivered in vitro or in vivo [75] by synthetically generated modified mRNAs. The cellular translation machinery is used to translate the mRNA in physiological states, and contrary to vectors of viral gene therapy, the lack of integration in the host genome makes it non-oncogenic [76, 77]. Therefore, in the future, treatment with modified synthetic mRNA will be more widely accepted.

mRNA vaccine technology, a new era in vaccinology has emerged as a promising platform for treating allergies, autoimmune illnesses, and cancers. This technology includes the usage of a nano-based nanocarrier that encapsulates an mRNA collection encoding the preferred antigens or epitopes to target specific tissues or cell types and set off immune tolerance [78, 79]. mRNA NPs such as lipid NPs (LNPs) are formulated with the use of ionizable cationic lipids (e.g., MC3), helper lipids (e.g., DSPC and LDL cholesterol), and PEG lipids, which wrap around mRNA molecules and form micelles that deliver and protect the mRNA molecule to the cytosol for protein expression. After the system of those lipids is determined, the lipid NPs can efficiently encapsulate mRNA molecules in a microfluidic device and securely deliver them to their goal cells. When mRNA LNPs enter cells by phagocytosis or endocytosis the mRNA can escape from endosomes and be translated to antigenic proteins or peptides [80]. mRNA synthesized after being taken up via APCs is translated to the interest peptide into the cytosol. This manufactured peptide, which cannot be recognized from the endogenous mRNA product, undergoes post-translational modifications, and parts of it are degraded by intracellular components. The MHC of APCs presenting these peptides can stimulate the host immune system, which leads to the induction of cancer-specific killer T cells as well as activated helper T lymphocytes and NK cells [17, 81]. MHC class I and II cross-present extracellular proteins for activation of CD4^+^ T cells. The CD4^+^ T cells are capable of co-activating protein-specific B cells, and B cells are also capable of co-activating CD4^+^ T cells after the B cell receptor induces antigen internalization [82]. Moreover, engineered mRNA constructs affected the production of pro-inflammatory cytokines such as IL-2, IL-7, IL-12, and IL-15. These pro-inflammatory cytokines increase the production of antigen-specific CD8^+^ cytotoxic T cells and the ratio of active CD8^+^ cells to immune suppressor regulatory T cells (T_Regs_) and result in the generation of memory T cells for an extended and lasting immune response [17, 83] (Fig. 3).

### Engineered mRNA for enhanced translation efficiency

**Fig. 4:**
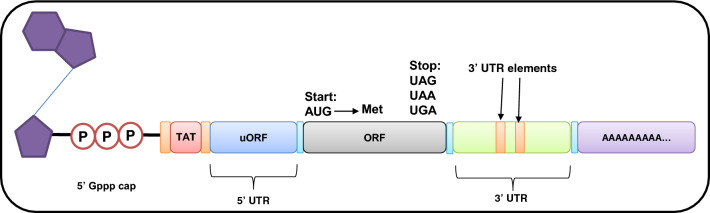
The schematic map of mRNA vaccine construct. The final construct of an mRNA vaccine includes an ORF, UTRs, a 5′ cap, and a poly (A) tail. The mRNA vaccines can be classified as self-amplifying mRNA (SAM) or non-replicating mRNA (NRM). Several features are similar between them, including a 5′ cap sequence, 5′ and 3′ UTRs, an ORF that contains coding sequence, and a 3′ poly (A) tail. In SAM, genetic replication machinery derived from flaviviruses and alphaviruses is the main difference between them. Created with BioRender.com

**Fig. 5:**
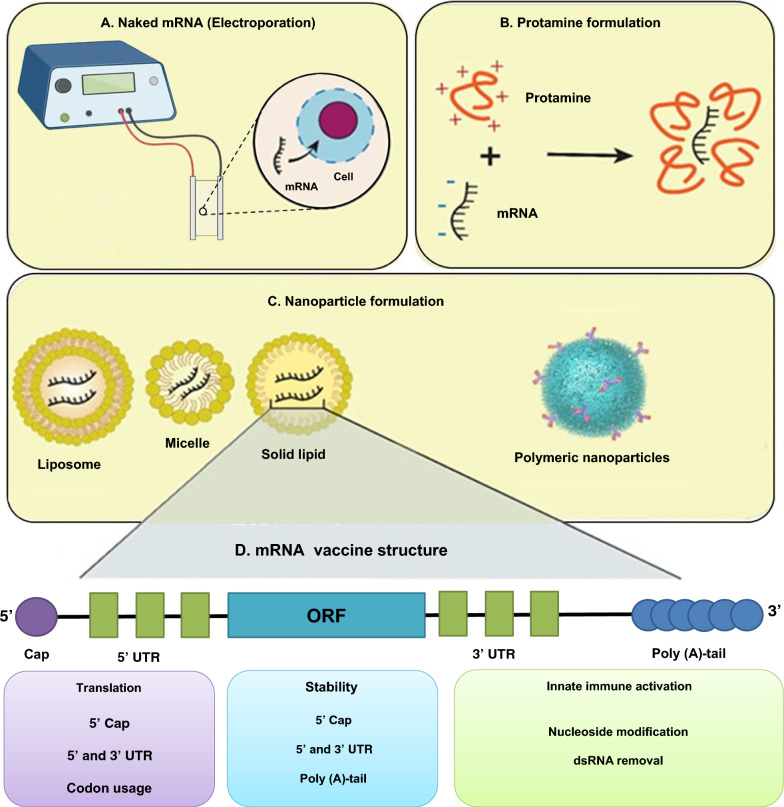
The vaccine efficacy increases by delivery systems selection and construct modifications. mRNA vaccines can be delivered using **A** naked mRNA, **B** protamine formulation lipid NPs, and **C** polymeric NPs. In addition to protecting mRNA from enzymatic degradation, delivery systems also protect it from extracellular ribonuclease degradation. Cationic lipids with positive charges regulate the location of mRNA at the negatively charged surface of cells. They also facilitate endocytosis and the escaping of endosomes. **D** The vaccine efficacy is influenced by the appropriate construct element selection and design. Created with BioRender.com

The design and stability of synthesized mRNA play a pivotal role in translation efficiency (Figs. 4 and 5). Consequently, optimizing the structural components within IVT mRNA is often essential for the consistent expression of target proteins at stable levels. Hence, to maintain a constant and stable expression of target proteins, it is beneficial to fine-tune the structural elements of IVT mRNA.

#### Cap

The 5′ cap of mRNA plays a crucial role in the translation process. In eukaryotes, it comprises 7-methylguanosine (m7G) bound to the 5′ end of mRNA via a 5′-5′-triphosphate bridge (ppp) (m7GpppN), offering numerous advantages for translation and mRNA preservation [84]. Two primary capping methods were employed for the synthesis of IVT mRNA. It can be achieved either through the utilization of enzymes involved in capping or by integrating a cap analog during transcription in test-tube (in vitro) [85].

#### Untranslated regions

The principal role of untranslated regions (UTRs) lies in the regulation of posttranscriptional gene expression, which has a substantial impact on mRNA bioactivity. The specific sequences within UTRs are able to interact with diverse regulatory proteins. In addition, the UTR length and the secondary structures are pivotal for improving translation efficiency [86, 87].

#### Open reading frame

To facilitate the expression of the desired protein, the open reading frame (ORF) furnishes essential genetic instructions for the translation process. Unlike various mRNA elements, an ORF does not primarily contribute to augmenting the expression level or stability of mRNA. However, it can exert influence over the translational functions and stability of mRNA, contingent upon its base composition [88].

#### Poly (A) tail

The final element responsible for modulating mRNA stability and translational activity is the poly (A) tail, which interacts with the poly (A) binding protein (PABP) and shields mRNA from degradation by 3′ to 5′ nucleases. Consequently, it is essential to maintain an appropriate length for optimal interaction with PABP and mRNA stability. Currently, a poly (A) tail length ranging from 64 to 150 nucleotides is considered the standard for achieving efficient translation [89, 90].

### In vitro transcription of mRNA

The initial step in IVT mRNA preparation involves crafting the DNA template, which necessitates a minimum of four components: the promoter of bacteriophage, ORF, UTR, and Poly (T). In the reaction solution, all four distinct ribonucleoside triphosphates (rNTPs), RNA polymerase, and the DNA template were incorporated. To obtain pure IVT mRNA, it is essential to remove non-reacted nucleotides, short oligonucleotides, enzymes, and extra salts. Purification can be achieved using mRNA purification kits in a laboratory setting or by using liquid chromatography techniques for large-scale IVT mRNA purification.

## Glioblastoma antigens and immune subtypes

**Fig. 6:**
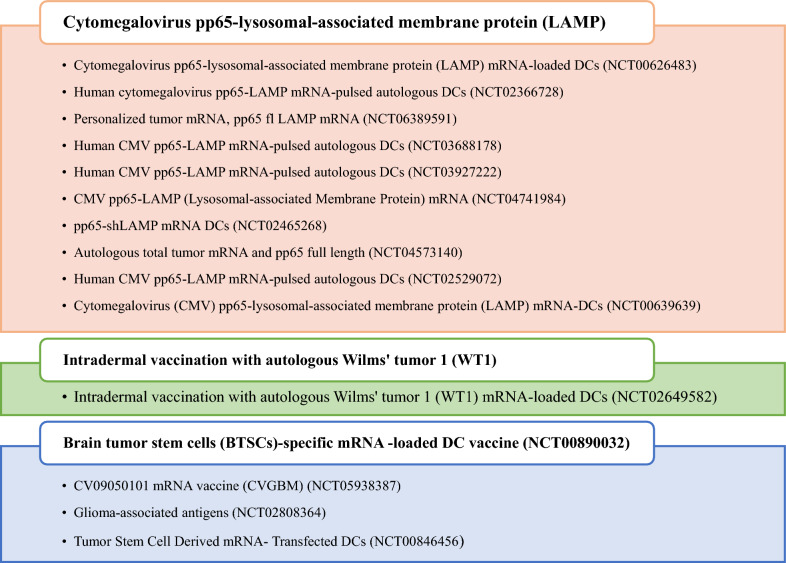
Current antigens for mRNA glioblastoma vaccines in clinical trials

The selection of antigens is of paramount importance in the development of a GBM vaccine. mRNA vaccines facilitate an indirect stimulation of the immune system, leading to an anti-tumor response. Traditional radiographic imaging methods do not consider this distinctive mechanism of action and may fail to provide an accurate representation of the actual clinical advantages of mRNA vaccines. Each patient’s unique condition and cancer type will require a different treatment plan and dosage for personalized mRNA cancer vaccines [91]. Novel biomarkers and tumor antigens that can accurately monitor the treatment response to these vaccines are needed to determine which mRNA cancer vaccines should advance beyond early phase trials and into larger phase III clinical trials [92]. Antigens that are specifically expressed by tumor cells are necessary for survival and are highly immunogenic [93]. Few antigens have been employed in cancer vaccines and have the aforementioned characteristics. Tumor antigens are divided into two types, tumor-associated antigens (TAAs) and tumor-specific antigens (TSAs), according to their tissue distribution, expression level, and central tolerance status, which are antigenic markers. TSAs are MAPs that are found only on cancer cells robust evidence indicates that anti-tumor immune responses potentiated via immune checkpoint therapy are directed against TSAs [94, 95]. However, the molecular landscape of actionable TSAs remains largely elusive [96]. As opposed to TSAs, TAAs are extensively used to produce vaccines for most cancers. Currently, 15 clinical trials are ongoing, and a list of current antigens targeting different GBM are summarized in Fig. 6.

TAAs are overexpressed in tumor cells but have low or silent expression levels in healthy cells [97]. T cells bind with high affinity to TAAs as self-antigens, and the majority of them are removed by peripheral and central tolerance mechanisms; therefore, a cancer vaccine containing these antigens needs to “break tolerance” [98]. TAAs exhibit differential expression in multiple GBM subtypes and are routinely employed as biomarkers for various malignancies. The origin, stage, grade, and even the examination method of the tumor can affect the expression of antigens [99]. Many antigens have been identified in GBM, including tumor-promoting and tumor suppressor proteins. Increasing evidence indicates that antigens with different functional proteins associated with GBM are numerous and play a critical role in GBM initiation, progression, and recurrence [100] (Table 2).

**Table 2:** Some GBM-associated antigens and GBM-associated antigens

Biomarkers	Description	Ref.
B7H3	Also known as CD276, a member of the B7 family of immune checkpoint proteins	[171]
IL-13Rα2	Interleukin-13 receptor alpha 2	[172]
HER2	Human epidermal growth factor receptor 2	[173]
EGFRvIII	Epidermal growth factor receptor variant III	[174]
EphA2	Erythropoietin-producing hepatocellular carcinoma A2	[175]
GLEA2	Glioma-expressed antigen 2	[176]
ARHGAP9	Rho GTPase activating protein 9	[105]
ARHGAP30	Rho GTPase activating protein 30	
CLEC7A	C-type lectin domain family 7	
MAN2B1	Mannosidase alpha class 2B member 1	
ARPC1B	Actin-related protein 2/3 complex subunit 1B	
PLB1	Phospholipase B1	
Survivin	Also called baculoviral inhibitor of apoptosis repeat-containing 5 or BIRC5	[177]
WT1	Wilms tumor protein	[177]
GD2	Ganglioside 2	[178]
PDGFRA	A transmembrane receptor containing five immunoglobulin-like domains and one tyrosine kinase domain	[179]
NKG2D	Natural Killer Group 2 member D	[180]
MET	Mesenchymal–epithelial transition factor	[181]
HK3	Hexokinase 3	[107]

Neo-antigens are cancer-specific antigens that are unique to each patient’s cancer. Since somatic mutations are created in the genome of tumor cells, they can be highly immunogenic and do not undergo central tolerance. As a result, a flexible and powerful vaccine technology is needed to create personalized neo-antigen vaccines [97]. Tumor neoantigens, in contrast to TAA, are ideal targets for immunotherapy because they can be recognized as non-self antigens; thus, they elicit stronger antitumor T cell responses [101]. Studies have shown that few neoantigens cause a strong immune response against the tumor [102]. Therefore, cancer patients’ neoantigens can be distinguished from normal proteins using strategies such as genome sequencing. Comparing the sequence of tumor cells and normal cells with advances in next-generation sequencing (NGS) technologies is a very cost-effective method and helps to identify cancer neoantigens [103].

The TAAs and tumor immune microenvironment changes have been identified in GBM [104]. As an example, in a study, some potential tumor antigens were identified for GBM vaccine development and development of a tool for predicting how GBM patients respond to vaccination. The expression profiles of GBM antigens were evaluated using gene expression profiling interactive analysis (GEPIA), and genetic alterations were analyzed using the cBioPortal program. The TIMER program was used to analyze the correlation between APC and antigens. Further clustering analysis was conducted using GBM RNA-seq data and corresponding clinical data from The Cancer Genome Atlas (TCGA) and the Chinese Glioma Genome Atlas (CGGA). The survival rate of GBM patients and the presence of APCs in GBMs were highly correlated with six overexpressed or mutated tumor antigens (ARPC1B, ARHGAP9, ARHGAP30, CLEC7A, PLB1, and MAN2B1). GBMs that belong to the IS3 subtype were found to respond better to vaccination than GBMs that belong to the IS1 subtype. The immune landscape among GBM patients was depicted by dimensional reduction using graph learning. Furthermore, WGCNA is capable of identifying immune-related genes as potential vaccine biomarkers [105]. In addition, to detect antigens useful in developing mRNA vaccines for GBM, researchers in another study assessed the immune subtypes of GBM to establish selection criteria for suitable vaccination candidate selection. In GBM, TP53, IDH1, C3, and TCF12 have been identified as mutated and overexpressed antigens associated with poor prognosis. Furthermore, the TCGA data consistently identified ten immune gene modules and four GBM immune subtypes (IS1–IS4). Molecular, cellular, and clinical characteristics differentiate the immunity subtypes. In contrast to IS2 and IS3, IS1 and IS4 were associated with an immune-activating phenotype and worse survival. All four immune subtypes expressed immune checkpoints and immunogenic cell death regulators [106]. Moreover, Ye et al.’s study identified the appropriate population for cancer vaccination through immune-phenotyping by identifying possible tumor antigens of GBM. The results showed that GBM patients with immune subtypes 1 and 2 had distinct clinical outcomes, indicating immune suppression and immune inflammation, respectively. GBM mRNA vaccination could be developed using ARPC1B and HK3 mRNA antigens, and patients in IS2 were considered the most suitable population [107].

## Modulation of mRNA immunogenicity

Several important innovations have recently boosted the development and design of mRNA vaccines, including technologies that produce and deliver high-quality mRNAs. Several technical obstacles have been overcome in the past, including stability, delivery, and immunogenicity [108].

### Introduction of modified nucleosides

**Fig. 7:**
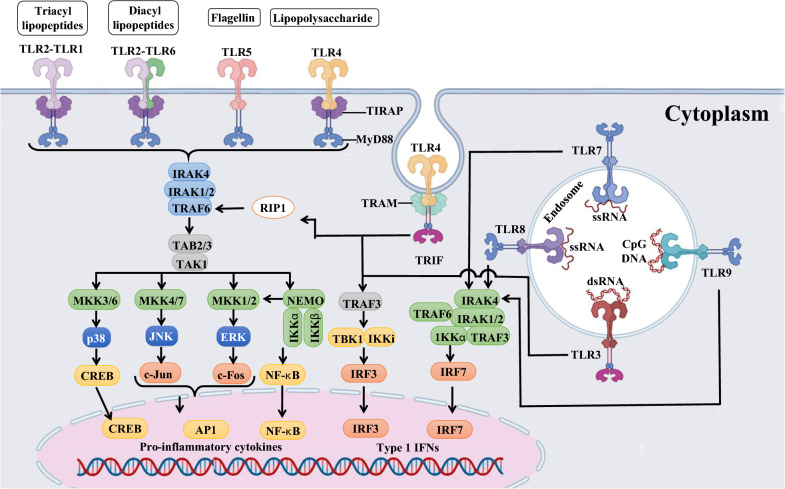
Overview of TLR-activating and the induced signaling pathway. Created with BioRender.com

The introduction of modified nucleosides may be a positive approach for modulating mRNA. The delivery of single-stranded mRNA molecules to cells exogenously is itself a PAMP aside from the double-stranded RNA (dsRNA) contaminants [109]. Several innate immune receptors recognize exogenous mRNA, including those on the cell surface, endosomes, and cytosol [109]. The toll-like receptors (TLRs) of innate immune cells can be activated by exogenous mRNA, especially TLR7 and TLR8. When incorporated into transcripts, certain naturally occurring modified nucleosides decrease TLR activation [74, 110] (Fig. 7). There are several examples, such as m5C, m6A, m5U, s2U, or pseudouridine that reduce the RNA-mediated immune response, resulting in a reduction of type I interferon (IFN) signaling while improving translational capacity and stability [72, 73, 111]. For instance, Karikó et al*.* found that nucleoside-modified mRNA translations is more efficient in vitro in primary DCs and in vivo in mice than unmodified mRNA [112].

### Regulation of self-adjuvant properties by purification of mRNA modulates

The dsRNA is commonly present in in-vitro transcription products of mRNA. Type I IFN production can be promoted by dsRNA, which mimics the replication intermediates of RNA viruses [113]. Pathogen-associated molecular patterns (PAMPs) in multiple cellular compartments sense dsRNA as a mimic of viral genomes and replication intermediates [114]. IVT mRNA contaminated with the dsRNA leads to robust type I IFN production. By increasing protein kinase R (PKR) and 2′-5′-oligoadenylate synthetase (OAS) expression and activation, mRNA translation is inhibited and ribosomal RNA and cellular mRNA are degraded [115]. It has been demonstrated that chromatographic methods (e.g., fast protein liquid chromatography and high-performance liquid chromatography) can effectively remove dsRNA from mRNA [116]. It is possible to increase mRNA translation in primary cells by 10–1000 times after purification while still maintaining relatively high cytokine production levels [117]. In other words, purifying IVT mRNA appropriately is critical to maximize protein production in DCs and avoid unwanted innate immune activation [109].

### Regulation of self-adjuvant properties by optimizing mRNA sequences

The innate immunity induced by mRNA sequences is a major obstacle to the development of safe and effective mRNA vaccines [108, 118]. Type I IFN can be produced by single-stranded RNA (ssRNA), which induces a wide range of IFN-stimulated genes (ISGs) to inhibit the translation of mRNA. Therefore, different characteristics of mRNA sequences must be optimized if mRNA vaccines are to be successful. The 5′-terminal cap (m^7^GpppN-, cap 0)-capped and uncapped mRNAs can be recognized by pattern recognition receptors (PRRs) and inhibit translation. Eukaryotic translation initiation factor 4E (eIF4E) is recruited by the 5′ cap to facilitate ribosome recognition and translation initiation [84, 119, 120]. Since different cap structures play a key role in cell recognition processes, it becomes clear that uncapped transcripts are inadequate representations of eukaryotic mRNAs, and it is essential to prepare correctly capped RNAs to evaluate mRNA function in cells [121]. It has also been demonstrated that modifying mRNA cap structure increases translational efficiency and stability [121, 122]. Based on the desired length of the capped RNA, a variety of capped RNAs can be produced using either a fully synthetic or enzymatic approach, ranging from a few nucleotides to authentic mRNAs (> 1000 nt) [121]. Post-translational capping enzymes are widely used in vitro, with the most common being the vaccinia capping enzyme (VCE) [123, 124]. The VCE consists of two subunits (D1 and D12). In addition to the triphosphatase, guanylyltransferase, and methyltransferase activities of D1, D12 plays a vital role in activating D1 [120].

Alterations in the ORF region may diminish the robust immune response triggered by the recognition of PRRs, while simultaneously increasing the translation efficiency of mRNA [117]. To improve translation efficiency and prevent innate immunity reactions due to PRR recognition, several approaches have been developed to modify ORF sequences. GC content and codon usage bias can be modified to regulate translation elongation rates, or codon usage bias can be modified to avoid secondary structures [125, 126]. Increased GC content can also be attributed to codon optimization using uridine depletion. Additionally, various strategies can be used for codon optimization, including using codons with a higher transfer ribonucleic acid (tRNA) abundance or using more frequent codons. In addition, the best pairs of codons can be used together to optimize the dicodon usage. Third, the ORF sequence can be modified to have the same ratio of codons as natural proteins found in the target species and cells. Although high translation rates are beneficial, not all proteins fold properly and effectively when translated at a high rate. Therefore, a moderate translation rate and high translation accuracy should be ensured by codon optimizations in the ORF [125, 127].

### Optimizing the mRNA immunogenicity with adjuvants

A mRNA vaccine system can be enhanced by adding adjuvants to improve immune response to antigens [128]. Some vaccine formulations incorporate adjuvants as exogenous materials, while others add them as exogenous materials. These include conventional adjuvants as well as novel adjuvant approaches that exploit the intrinsic immunogenicity of mRNA or its ability to encode immune-modulating proteins [109]. Despite the inherent self-adjuvant properties of naked IVT mRNA, additional materials such as protamine, CpG motifs, and poly I:C RNA can be combined with unbounded IVT mRNA to enhance the ability of an mRNA vaccine to stimulate adaptive immunity as well [129].

Adjuvant effects can also be increased by some mRNA delivery systems, including cationic lipids and protamine [117]. It has been demonstrated that MF59 is a useful adjuvant that enhances immunogenicity. A cationic nanoemulsion (CNE) delivery system is described for the delivery of a self-amplifying mRNA vaccine. The proprietary adjuvant MF59 of Novartis is used in these nonviral delivery systems, and the system has also demonstrated enhanced immunogenicity and efficacy in animal models [130, 131]. Furthermore, the effectiveness of a cationic lipid 1,2-dioleoyl-3trimethylammonium-propane/1,2-dioleoyl-sn-glycero-3-phosphoethanolamine (DOTAP/DOPE), as a novel vaccine strategy for antigen-encoding mRNA, was investigated. Results showed that antigen-encoding mRNA complexed with DOTAP/DOPE displayed immune activating properties by releasing type I IFN and recruiting monocytes to drain the lymph nodes. It is demonstrated that type I IFN inhibits the expression of DOTAP/DOPE complexed antigen-encoding mRNA and subsequent immune responses to antigens. Hence, cationic lipids may enhance the effectiveness of mRNA vaccines by strengthening the adjuvant effect [132]. In addition, studies have shown that RNA condensed on protamine can be protected from degradation by RNase. In addition to activating TLR7 and TLR8, such complexes are danger signals that activate T-help 1 cells (Th1) [128].

## mRNA vaccines against glioblastomas in clinical trials

**Fig. 8:**
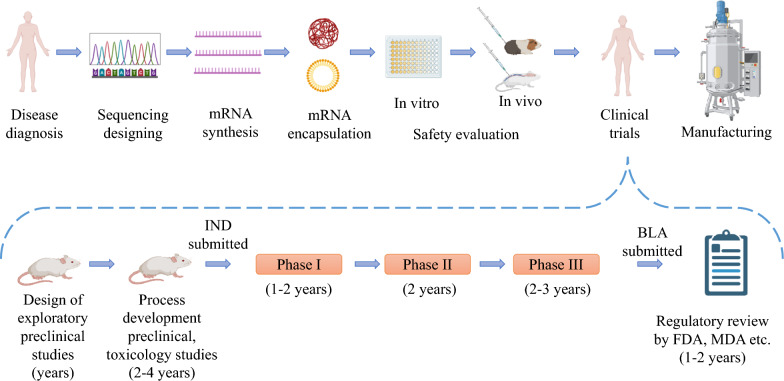
The process of creating novel mRNA vaccines from sequence design to large-scale production. The process of creating novel mRNA drugs from sequence design to clinical translation is shown in the upper part. The lower part shows that mRNA drug can progress to pre-clinical tests and clinical trials after design and production. During this process an investigational new drug (IND) application is filed and the vaccine candidate then enters phases of clinical trials. If, while phase III trials are completed, the predetermined endpoints have been met, a biologics license application (BLA) is filed, reviewed by regulatory organizations, and ultimately the vaccine is licensed. Created with BioRender.com

Nowadays, sixteen clinical trials have been registered investigating the use of mRNA vaccines for the treatment of GBM. Among them, three trials have published their results, which will be discussed in detail below. All trials are summarized in Table 3 and Fig. 8.

**Table 3:** Selected therapeutic mRNA-based vaccines for glioblastomas in clinical trials

NCT number	Study title	Study status	Conditions	Interventions	Results	Current responsible party	Study phase	Enrollment
NCT00846456	Safe study of DC-based therapy targeting CSCs in GBM	Completed	GBM Brain tumor	BIOLOGICAL: DC vaccine with mRNA from CSCs	Progression-free survival: 694 days (vaccinated) vs. 236 days (controls, p = 0.0018). OS trend: 759 days (vaccinated) vs. 585 days (controls, p = 0.11). Specific T-lymphocyte proliferation in response to tumorsphere lysate, hTERT, or survivin peptides. MRI findings showed initial increase in contrast-enhancing lesions followed by a significant reduction	Steinar Aamdal, Oslo University Hospital	1 and 2	20
NCT02529072	Nivolumab with DC vaccines for recurrent brain tumors	Completed	Malignant GBM Astrocytoma GBM	DRUG: nivolumab BIOLOGICAL: DC Group I: nivolumab 3 mg/kg IV every 2 weeks for 8 weeks Group II: nivolumab 3 mg/kg IV + DC vaccine every 2 weeks for 3 doses, then surgery, followed by nivolumab every 2 weeks and DC vaccine monthly for 5 more doses	Group II showed a longer median OS (15.3 months) compared to Group I (8.0 months) Progression-free survival was also longer in Group II (6.3 months) compared to Group I (4.3 months) Serious adverse events were more common in Group II (66.67%) compared to Group I (33.33%)	Gary Archer, Duke University	1	6
NCT02808364	Personalized cellular vaccine for recurrent GBM (PERCELLVAC2)	Completed	GBM	Personalized cellular vaccine consisting of mRNA tumor antigen pulsed autologous DCs administered biweekly	Antigen-specific CD4^+^ and CD8^+^ T cell responses were induced without obvious autoimmune adverse events	Jian Zhang, Guangdong 999 Brain Hospital	1	10
NCT00626483	Basiliximab in treating patients with newly diagnosed GBM undergoing targeted immunotherapy and TMZ-caused lymphopenia	Completed	Malignant neoplasms brain	Basiliximab [20 mg or 40 mg (two doses per cycle)] Temozolomide (TMZ) [75 mg/m^2^ (during RT), 150–200 mg/m^2^ (post-RT)] RNA-loaded DC vaccine (2 × 10^7^ cells per dose, administered monthly) GM-CSF (administered intradermally with each vaccine) Radiotherapy (RT) (stereotactic, concurrent with initial TMZ course)	Not reported	Gary Archer, Duke University	1	34
NCT02366728	DC migration study for newly diagnosed GBM	Completed	GBM Astrocytoma, grade IV Giant cell GBM GBM	Group I: 1 × 10^6^ unpulsed DCs (0.4 mL) one side of groin + 2 × 10^7^ CMV pp65-LAMP DCs (up to 10 vaccines) + 111In-labeled DCs (4th vaccine) + temozolomide (150–200 mg/m^2^/d) + Saline (0.4 mL) opposite groin Group II: Td toxoid (1 flocculation unit, 0.4 mL) one side of groin + 2 × 10^7^ CMV pp65-LAMP DCs (up to 10 vaccines) + 111 In-labeled DCs (4th vaccine) + temozolomide (150–200 mg/m^2^/d) + Saline (0.4 mL) opposite groin Group III: basiliximab (20 mg I.V. pre-vaccines) + Td toxoid (1 flocculation unit, 0.4 mL) one side of groin + 2 × 10^7^ CMV pp65-LAMP DCs (up to 10 vaccines) + temozolomide (150–200 mg/m^2^/d) + saline (0.4 mL) opposite groin	Group I: 25 participants, 23 completed, 4 not completed; Median OS: 16 months; median progression-free survival: 6.5 months; % of 111 In-labeled DCs migrating to inguinal lymph nodes: 6.0% Group II: 27 participants, 27 completed, 1 not completed; median OS: 20 months; median progression-free survival: 6.7 months; % of 111In-labeled DCs migrating to inguinal lymph nodes: 9% Group III: 8 participants, 8 completed, 1 not completed; median OS: 19 months; median progression-free survival: 7.1 months; % of 111In-labeled DCs migrating to inguinal lymph nodes: not collected	Mustafa Khasraw, Duke University	2	64
NCT00890032	Vaccine therapy in treating patients undergoing surgery for recurrent GBM	Completed	Recurrent CNS neoplasm	BIOLOGICAL: BTSC mRNA-loaded DCs Initial dose: 2 × 10^6^ BTSC mRNA-loaded DCs Escalation: 5 × 10^6^, 2 × 10^7^ per vaccination	Not reported	John Sampson, Duke University	1	50
NCT00639639	Vaccine therapy in treating patients with newly diagnosed GBM	Completed	Malignant neoplasms of brain	BIOLOGICAL: tetanus toxoid BIOLOGICAL: therapeutic autologous DCs BIOLOGICAL: therapeutic autologous lymphocytes	Not reported	Gary Archer, Duke University	1	42
NCT04741984	Monocyte antigen carrier cells for newly diagnosed GBM	Withdrawn	GBM	BIOLOGICAL: MT-201-GBM monocyte vaccine [monocytes isolated from patient’s leukapheresis loaded with CMV pp65-LAMP (lysosomal-associated membrane protein) mRNA (messenger ribonucleic acid)]	Not reported	Michael Gunn, Duke University	1	–
NCT03927222	Immunotherapy targeted against CMV in patients with newly diagnosed WHO grade IV unmethylated GBM	Terminated	GBM	BIOLOGICAL: human CMV pp65-LAMP mRNA-pulsed autologous DCs containing GM CSF (2 × 10^7^ cells, intradermally, bilaterally at groin site) DRUG: temozolomide (100 mg/m2/day for 21 days post-RT) BIOLOGICAL: tetanus–diphtheria toxoid (Td) (0.5 mL intramuscularly, 0.4 mL intradermally) BIOLOGICAL: GM-CSF (250 mcg, reconstituted in 0.5 mL of sterile water) BIOLOGICAL: 111-indium-labeling of cells for in vivo trafficking studies (50 μCi/5 × 10^7^ DCs labeled)	Not reported	Mustafa Khasraw, Duke University	2	6
NCT04911621	Adjuvant DC immunotherapy for pediatric patients with high-grade GBM or diffuse intrinsic pontine GBM	Active_not_Recruiting	High-grade GBM diffuse intrinsic pontine GBM	BIOLOGICAL: DC vaccination + TMZ-based chemoradiation BIOLOGICAL: DC vaccination + conventional next-line treatment	Not reported	University Hospital, Antwerp	1 and 2	10
NCT02465268	Vaccine therapy for the treatment of newly diagnosed GBM	Active_not_Recruiting	GBM|GBM Malignant GBM Astrocytoma, grade IV GBM	Experimental: pp65-shLAMP DC with GM-CSF and Td Experimental: pp65-flLAMP DC with GM-CSF and Td Placebo comparator: unpulsed PBMC and saline	Not reported	University of Florida	2	175
NCT03688178	DC migration study to evaluate T_Reg_ depletion In GBM patients with and without Varlilumab	Active_not_Recruiting	GBM	Group 1: DC vaccine (unpulsed DC pre-conditioning), temozolomide, up to 10 DC vaccines Group 2: DC vaccine (Td pre-conditioning), temozolomide, up to 10 DC vaccines Group 3: DC vaccine + varlilumab (Td pre-conditioning), temozolomide, up to 10 DC vaccines, and varlilumab infusions	Not reported	Annick Desjardins, Duke University	2	43
NCT04573140	A study of RNA-lipid particle (RNA-LP) vaccines for newly diagnosed pediatric high-grade GBMs (pHGG) and adult GBM	Recruiting	Adult GBM	BIOLOGICAL: autologous total tumor mRNA and pp65 full length lysosomal associated membrane protein mRNA loaded DOTAP liposome vaccine administered intravenously (RNA loaded lipid particles, RNA-LPs)	Not reported	University of Florida	1	28
NCT05938387	Safety and tolerability of CVGBM in adults with newly diagnosed MGMT-unmethylated GBM or astrocytoma	Recruiting	GBM	Dose escalation (part A): – Dose level -1: CVGBM 6 μg – Dose level 1: CVGBM 12 μg – Dose level 2: CVGBM 25 μg – Dose level 3: CVGBM 50 μg – Dose level 4: CVGBM 100 μg Dose expansion (part B): – CVGBM 100 μg (RDE^a^)	Not reported	CureVac	1	54
NCT03396575	Brain stem GBMs treated with adoptive cellular therapy during focal radiotherapy recovery alone or with dose-intensified TMZ	Recruiting	Diffuse intrinsic pontine GBM (DIPG) Brain stem GBM	BIOLOGICAL: TTRNA-DC vaccines with GM-CSF BIOLOGICAL: TTRNA-xALT DRUG: cyclophosphamide + fludarabine lymphodepletive conditioning|DRUG: dose-intensified TMZ DRUG: Td vaccine BIOLOGICAL: autologous HSC	Not reported	University of Florida	1	21
NCT02649582	Adjuvant DC-immunotherapy plus TMZ in GBM patients	Recruiting	GBM	BIOLOGICAL: DC vaccine plus TMZ chemotherapy (150–200 mg/m^2^/d temozolomide)	Not reported	Zwi Berneman, University Hospital, Antwerp	1 and 2	20

GBM: glioblastoma; DCs: dendritic cells; WHO: World Health Organization; GM-CSF: granulocyte-macrophage colony-stimulating factor; CMV: cytomegalovirus; T_Reg_: regulatory T cells; PD-L1: programmed death-ligand 1; LPs: lipid nanoparticles; CSCs: cancer stem cells; BTSCs: brain tumor stem cells; Td: tetanus–diphtheria toxoid; DIPG: diffuse intrinsic pontine GBM; TMZ: temozolomide; HSCs: hematopoietic stem cells

A study (NCT00846456) conducted by Oslo University Hospital assessed the safety and scientific validity of DC-based therapy targeting GBM stem cells (GSCs). This Phase I/II trial, which began in January 2009 and concluded in February 2013, involved 20 participants who received intradermal injections of transfected DCs. The intervention included a DC vaccine containing mRNA derived from cancer stem cells (CSCs) and was administered in combination with standard GBM therapy. The primary objective was to evaluate the feasibility and potential adverse effects of this therapy, with secondary outcomes focusing on immunological responses, time to disease progression, and OS over 5 years. Eligibility criteria included patients aged 18–70 with histologically confirmed Grade IV GBM and suitability for combined radiation and chemotherapy (“Stupps regimen”). Exclusion criteria covered various factors, such as tumor location, prior malignancy, chronic infections, and pre-existing cardiac or medical conditions that might limit activity or survival. This study is the first to explore the safety, potential efficacy, and feasibility of active immunotherapy targeting GSCs, specifically in a well-characterized population of CSCs in solid tumors. The researchers used sphere-forming assays to demonstrate that sphere-forming ability is a strong negative prognostic indicator in GBM patients. Autologous GSC cultures were successfully created from 32 GBM biopsies, with a median patient survival of 271 days when GSC cultures were formed. The vaccine production process involved GSC culture, RNA isolation, cDNA generation, and DC transfection with mRNA constructs. Patients who received DC vaccinations exhibited specific T-lymphocyte proliferation responses against GSC lysates, hTERT, and survivin peptides. Safety monitoring revealed manageable adverse events, comparable to standard therapy, and immune responses were observed despite the lymphopenia induced by TMZ treatment. MRI evaluations indicated initial tumor growth followed by a significant reduction in contrast-enhancing lesions. Compared to historically matched controls, vaccinated patients experienced longer progression-free survival (694 vs. 236 days), suggesting a potential therapeutic benefit. However, the study had limitations, including a small sample size and challenges in immune monitoring due to the limited GSC material. Despite these limitations, the study suggests that inducing a GSC-specific immune response without serious adverse effects is feasible, supporting the therapeutic potential of targeting CSCs in GBM and possibly other solid tumors [133].

In a 2015 study, researchers conducted a phase II clinical trial aimed at enhancing the efficacy of DC vaccines for GBM treatment by pre-conditioning vaccination sites with tetanus/diphtheria (Td) toxoids. The trial involved randomizing GBM patients to receive either Td pre-conditioning or unpulsed, mature DCs before receiving bilateral vaccinations with cytomegalovirus (CMV) phosphoprotein 65 (pp65) RNA-pulsed DCs. The results showed that DC migration to vaccine site draining lymph nodes (VDLNs) was significantly improved in patients pretreated with Td, which correlated with improved PFS and OS compared to those receiving DCs alone. Median PFS and OS were significantly higher in the Td-treated group, with several patients surviving longer than 36.6 months without progression. Notably, Td pre-conditioning significantly increased the patients’ survival, demonstrating its potential as an adjunctive treatment against cancer. This improvement was linked to increased migration of DCs to VDLNs, with correlations observed between DC migration and both PFS and OS. The pre-conditioning with Td, known to elicit CD4^+^ T cell responses, was also associated with an increase in pp65-specific immune responses, further bolstering its effectiveness. In addition, parallel experiments in mice mirrored these clinical findings, demonstrating that Td pre-conditioning led to amplified DC migration to VDLNs, correlating with suppressed tumor growth. The mechanistic understanding revealed that the increased migration was related to C-C motif chemokine ligand 3 (CCL3 or macrophage inflammatory protein-1α, MIP-1α), a chemokine found to be elevated in both patients and mice following Td pre-conditioning. Further experiments pinpointed the necessity of CCL3 and Td-activated CD4^+^ T cells for heightened DC migration, substantiating the pivotal role of these factors in enhancing antitumor responses. These findings not only highlight the significance of pre-conditioning in augmenting DC vaccines but also underscore the potential of DC migration as a predictive biomarker for immunotherapy studies [134].

In a clinical trial involving a case of GBM, a combination of immunotherapy was administered alongside DC vaccines, anti-programmed death-1 (anti-PD-1) therapy, poly I:C, and cyclophosphamide, all integrated with standard chemoradiation treatment. The patient achieved a disease-free status for 69 months. The DC vaccines were formulated with tumor antigens, which included mRNA-neoantigens, mRNA-TAAs, and tumor lysates oxidized with hypochlorous acid (HOCl). Furthermore, the mRNA-TAAs were enhanced through a unique TriVac approach that combines TAAs with a destabilization domain and incorporates them into full-length lysosomal-associated membrane protein-1, thereby improving the presentation of antigens by MHC class I and II molecules.

In a clinical trial, a GBM case with combination immunotherapy along with DC vaccines, anti-programmed death-1 (anti-PD-1) and poly I:C and cyclophosphamide that was integrated with standard chemoradiation therapy, and the patient remained disease-free for 69 months. The patient received DC vaccines loaded with tumor antigens, including mRNA-neoantigens, mRNA-TAA, and hypochlorous acid (HOCl)-oxidized tumor lysates. In addition, mRNA-TAAs have been modified with a singular TriVac era that fuses TAAs with a destabilization domain and inserts TAAs into full-length lysosomal-associated membrane protein-1 to enhance MHC-I and II antigen presentation [135].

The clinical trial “AVeRT” (NCT02529072) investigated the safety and efficacy of combining nivolumab, an immune checkpoint inhibitor, with DC vaccines (Human CMV pp65-LAMP mRNA-pulsed autologous DCs) in treating recurrent Grade III and IV GBM. This open-label, randomized, interventional trial followed a parallel assignment model, dividing participants into two groups to assess the combination therapy’s safety, defined by the incidence of unacceptable toxicities, including severe adverse events and complications post-surgery. In Group I, patients received nivolumab at a dose of 3 mg/kg intravenously every 2 weeks for 8 weeks before undergoing surgical resection. Post-surgery, these patients continued treatment with nivolumab and received DC vaccines biweekly for a total of three doses, followed by additional nivolumab and monthly DC vaccinations for five more doses. Treatment with nivolumab continued every 2 weeks until disease progression. In Group II, patients initially received their fourth cycle of nivolumab, followed by the combined treatment of nivolumab and DC vaccines every 2 weeks for a total of three doses, then underwent surgery. After surgery, these patients resumed biweekly nivolumab and monthly DC vaccinations for five more doses, with nivolumab continuing every 2 weeks until disease progression. The trial enrolled six participants, all of whom completed the study. The participants in both groups were male, with a mean age of 52.9 years in Group I and 61.2 years in Group II. The primary outcomes focused on safety and evaluating toxicity during treatment, while secondary outcomes examined OS and PFS. The median OS was estimated at 8.0 months for Group I and 15.3 months for Group II, with a median PFS of 4.3 months for Group I and 6.3 months for Group II. Serious adverse events included hydrocephalus in one patient from Group I and various complications such as wound infections and falls in Group II.

An oncology study, NCT00626483, explored the use of basiliximab in treating newly diagnosed GBM patients undergoing targeted immunotherapy following TMZ-induced lymphopenia by Gary Archer from Duke University. This study aimed to determine whether basiliximab inhibited the recovery of T-regulatory cells after therapeutic TMZ-induced lymphopenia. A combination of basiliximab with CMV pp65-lysosomal-associated membrane protein mRNA-loaded DCs and GM-CSF was investigated to determine its impact on the immune response against GBM. The trial design involved multiple assessments, including an evaluation of the safety of basiliximab, its effect on immune responses, alterations in immune cell profiles, progression-free survival rates, and immune cell infiltration in recurrent tumors. The study, which began on April 24, 2007, and ended on July 6, 2016, was conducted on 34 participants and explored the interaction between basiliximab, DC vaccines, and TMZ in treating GBM patients. The administration of daclizumab, coupled with a vaccination regimen involving CMV pp65 RNA-loaded DCs, adoptive transfer of naive lymphocytes, and subsequent DC vaccinations during TMZ cycles, showed promising results. After TMZ treatment, patients exhibited an increased frequency of immunosuppressive T_Regs_, which reduced following daclizumab administration, reflecting trends observed in preclinical models. Notably, the treatment did not hinder immune response enhancement, as four of six patients displayed an increase in pp65-specific T cells post-vaccination. Additionally, the treatment combination demonstrated good tolerability without adverse events related to immunotherapy; intriguingly, a majority of patients showed progression-free survival exceeding 24 months, underscoring the potential of this approach for further exploration in larger clinical trials. These findings are in the same line with preclinical studies, suggesting that the use of anti-IL-2Rα mAb in TMZ-treated GBM patients selectively enhances vaccine-driven antitumor immunity by reducing immunosuppressive T_Regs_ [136].

A clinical trial, NCT00639639, on vaccine therapy for treating patients with newly diagnosed GBM multiforme, aimed to evaluate the safety and efficacy of utilizing vaccines to stimulate the body’s immune response against tumor cells in patients with GBM multiforme. The study was completed by Gary Archer from Duke University, in collaboration with the National Cancer Institute (NCI). This interventional clinical trial enrolled 42 participants, utilizing a randomized phase I/II design to assess the impact of vaccine therapy in conjunction with radiation and chemotherapy, particularly TMZ-induced lymphopenia recovery. The primary objective of this study was to determine whether DCs containing CMV pp65-LAMP mRNA with or without autologous lymphocyte transfer were feasible and safe. Secondary objectives included evaluating humoral and cellular immune responses, time to progression, and the differential ability of labeled DCs to track the lymph nodes under various conditions. Eligibility criteria included individuals aged 18 or older with WHO Grade IV GBM, a KPS > 80%, and a Curran Group status of I-IV. Exclusion criteria included such factors as leptomeningeal or multicentric disease, prior anti-tumor therapy, pregnancy, continuous corticosteroid use, active infection, immunosuppressive disease, and previous inguinal lymph node dissection. The trial commenced on February 6, 2006, and ended on April 15, 2017. In this phase I trial, the aim was to assess the safety and effectiveness of a novel approach: using pp65-specific DCs combined with GM-CSF after dose-intensified TMZ (DI-TMZ) in newly diagnosed GBM patients. The trial involved 11 patients who received DI-TMZ followed by at least three vaccinations of pp65-DCs combined with GM-CSF. The study evaluated the immune responses targeting pp65 and their impact on long-term PFS and OS. The results showed a significant increase in pp65-specific immune responses after DI-TMZ and three doses of pp65-DCs. Interestingly, despite an increase in T_Regs_ following DI-TMZ, patients who received pp65-DCs demonstrated extended PFS and OS, surpassing predicted outcomes and matched historical controls. Notably, four patients remained progression-free at 59–64 months after diagnosis. This study suggests that despite increased T_Reg_ levels post DI-TMZ, patients who received pp65-DCs exhibited expanded antigen-specific immunity and prolonged survival, confirming earlier studies targeting CMV in GBM. This study demonstrates the potential of CMV-targeted immunotherapy to improve outcomes for newly diagnosed GBM patients [137].

In the clinical trial with ClinicalTrials.gov Identifier NCT02808364, titled “Personalized Cellular Vaccine for Recurrent GBM (PERCELLVAC2),” conducted by Guangdong 999 Brain Hospital in collaboration with Zhuhai Trinomab Pharmaceutical Co., Ltd., and other partners, the primary goal was to explore the potential of personalized immune cell-based therapy for recurrent GBM, a cancer known for its limited treatment options and poor prognosis. The study, initiated on March 1, 2016, and completed on June 30, 2019, enrolled ten participants who had undergone tumor resection and met specific inclusion criteria, such as a Karnofsky Performance Status (KPS) of 70 or higher. The trial sought to assess the safety and efficacy of personalized DC vaccines pulsed with TAAs derived from the patient’s tumors. The trial involved several key steps: after tumor resection, GBM-associated antigens were identified and used to generate IVT mRNA for pulsing the DCs. Patients received biweekly vaccinations with these personalized DCs, and their T cell responses to the tumor antigens were closely monitored. The treatment regimen also included low-dose cyclophosphamide, poly I, imiquimod, and anti-PD-1 antibodies to enhance the immune response. The study’s findings revealed that among the ten patients treated, seven showed specific CD4^+^ and/or CD8^+^ T-cell responses to the TAAs, which was indicative of the vaccines’ ability to stimulate an immune response. Importantly, no severe adverse events (Grade III/IV) were reported, suggesting that the treatment was well-tolerated. The survival outcomes were particularly notable: the median survival time for patients with advanced lung cancer was 17 months, and for those with GBM, it was 19 months, compared to 7 and 11 months, respectively, in a control group receiving standard treatments at the same institution [138].

## Safety aspects

No mRNA vaccine has been approved all over the world before 2020. As the SARS-CoV-2 pandemic continues, safety concerns have grown more important after licensing several mRNA vaccines against it in 2020. Various mRNA vaccines have now been tested in phase I through IIb clinical trials, and it has been established that they are safe and generally accepted [108].

Current preventive vaccinations must adhere to stringent safety standards since they are administered to healthy individuals. In contrast to other vaccine platforms, such as inactivated virus, viral vectors, live virus, and subunit protein vaccines, the manufacture of mRNA does not necessitate the use of hazardous chemicals or cell cultures that may become contaminated. Additionally, there are few possibilities for contaminating microorganisms to enter due to the quick mRNA production. Potential risks of infection or incorporation of the vector into the host cell DNA for mRNA in vaccinated individuals are not an issue. Due to the factors mentioned stated above, mRNA vaccines have been regarded as a generally safe vaccination formulation [109].

Among the potential safety issues, which will probably be investigated in future preclinical and clinical studies, are immunogen expression, biodistribution and persistence, local and systemic inflammation, any delivery system non-native nucleotides, and potential toxic efficacy of the component and stimulation of auto-reactive antibodies. One probable issue is that some platforms of mRNA-based vaccines prompt strong responses of type I IFN, which has been linked to both inflammation and autoimmune. Therefore, identifying those with a higher risk of autoimmune responses before mRNA vaccines may need appropriate safeguards. In addition, another safety risk is extracellular RNA during mRNA vaccine administration. Extracellular naked RNA increases the permeability of densely endothelial cells, which may help explain why edema occurs. Extracellular RNA increases blood coagulation and development of pathogenic thrombus, according to different research. Thus, due to various mRNA methods and delivery systems being used for the first time in people and being tested in larger patient groups, safety will need to be continually evaluated [109, 139].

## Regulatory aspects

Over the last few years, clinical trials for human mRNA vaccines and marketing authorization applications have increased significantly, and this trend is expected to continue. Preclinical and clinical findings indicating biodistribution and durability in mice, animal model protection (ferrets), and local reactogenicity, immunogenicity, and toxicity in humans were highlighted in a recent study of an mRNA vaccine against the influenza virus. As mRNA products gain prominence in the vaccination sector, it is expected that precise guidelines will be produced, outlining the need for developing and testing novel mRNA vaccines [140]. WHO is attempting to convene international discussions between producers, regulators, and vaccine developers to assess existing research evidence, address major concerns, and create consensus on science and technology expectations for safeguarding the efficacy, safety, and quality of mRNA vaccines [109, 141]. Moreover, the FDA and the European Medicines Agency (EMA) have not guided mRNA vaccination preparations. On the other hand, the growing number of clinical studies conducted under EMA and FDA supervision demonstrates that regulators have considered the methodologies presented by different organizations to establish that products are safe and suitable for testing in humans. Because mRNA falls within the broad vaccination category of genetic immunogens, many of the guiding concepts developed for DNA vaccines and gene therapy vectors may be used for mRNA with modest modifications to represent the specific properties of mRNA [109, 142, 143].

## Therapeutic considerations and challenges and limitations

Despite the enthusiasm in the mRNA-based immune-oncology field, challenges persist before clinical implementation, particularly in the context of GBM [117]. The effectiveness of mRNA vaccines against gliomas remains uncertain, largely due to the tumor’s heterogeneity and its immunosuppressive environment.

### Tumor heterogeneity

A major obstacle in using mRNA vaccines for GBM treatment lies in the tumor’s heterogeneity. GBM tumors consist of a complex mix of cell types, each potentially harboring distinct genetic mutations and expressing varying surface antigens. This diversity poses a significant challenge for developing a universal vaccine that can effectively target all tumor cells, as mRNA vaccines depend on the immune system’s ability to recognize and attack specific TAAs, which may not be consistently expressed across all cancer cells. GBM cells do express a range of TSAs and TAAs, both of which are crucial for vaccine design. TSAs are exclusively found in tumor cells and are often unique to individual patients, resulting from genetic mutations that produce novel peptide fragments (neoantigens), abnormal post-translational modifications, or viral infections. In contrast, TAAs are more commonly found in tumor cells, but can also be found in normal tissues. There is a risk that targeting these antigens could inadvertently trigger an immune response against normal tissues, leading to autoimmune complications. However, clinical trials have shown a more promising immune response to TAAs than to TSAs [8, 144, 145].

### Immunosuppressive microenvironment

The TME in GBM plays a critical role in shaping the efficacy of mRNA vaccines and poses significant challenges due to its complexity and heterogeneity. GBM tumors are characterized by a diverse molecular and cellular landscape, with tumor stem cells (TSCs) contributing to the formation of distinct cellular niches within the tumor. These TSCs, similar to normal stem cells, possess the ability to self-renew and differentiate into multiple cell types, further complicating the uniform targeting of tumor cells by mRNA vaccines. The variability of TSC populations in different GBM tumors exacerbates this challenge, as these cells often express higher levels of drug resistance proteins and anti-apoptotic genes, making them more resistant to treatments, including immunotherapies. In addition to intrinsic cellular diversity, the TME in GBM significantly influences the immune response. The interaction between malignant cells and the surrounding microenvironment facilitates tumor growth and impedes effective immune surveillance. Tumor-associated myeloid cells (TAMCs), including macrophages, microglia, and MDSCs, are prominent within the GBM TME. These immune cells typically adopt an immunosuppressive phenotype, secreting factors such as interleukin-10 (IL-10) and transforming growth factor-β (TGF-β), which suppress T cell activation and infiltration. In addition, TAMs in GBM often lack critical costimulatory molecules necessary for lymphocyte activation and instead, upregulate immunosuppressive ligands such as B7-H1 and Fas ligand. This immunosuppressive milieu within the TME not only hampers the efficacy of the immune system’s natural response to the tumor, but also poses a significant barrier to the success of mRNA vaccines, which rely on a robust and active immune response to target and eliminate tumor cells [146–149].

### Blood–brain barrier

The BBB presents a formidable challenge to the treatment of GBM with mRNA vaccines. This complex neurovascular structure, consisting of endothelial cells, pericytes, astrocytes, microglia, and smooth muscle cells, serves as a protective shield for the brain by tightly regulating the entry of substances. While the BBB is critical for maintaining neural homeostasis and protecting the brain from toxins, it also severely limits the delivery of therapeutic agents to brain tumors. Molecules must possess certain properties, such as lipophilicity and a molecular weight below 500 Da, to cross the BBB by simple diffusion. However, the impermeability of the BBB limits the effectiveness of many potential treatments, including mRNA-based therapies. Overcoming this challenge necessitates sophisticated nanoscale formulations that can navigate the BBB to specifically target GBM cells while minimizing adverse effects on healthy brain tissue [150–153].

In the context of GBM, the immune privilege of the central nervous system (CNS) further complicates the development of effective mRNA vaccines. This immune privilege results from the combined effects of the BBB, the paucity of MHC class II expressing antigen presenting cells, and the unique metabolic environment of the CNS. These factors create a formidable barrier to immune responses in the brain. Innovative approaches are being explored to overcome these barriers. For example, enhancing lymphangiogenesis in the brain with mRNA-encoded VEGF-C nanoparticles has shown promise in preclinical models. This strategy not only improves T-cell recruitment to GBM tumors, but also facilitates tumor antigen delivery to cervical lymph nodes, potentially enhancing the efficacy of mRNA vaccines when combined with immune checkpoint inhibitors. However, these approaches are still in the experimental stage, and the challenge of effectively delivering and activating mRNA vaccines in the highly protected CNS environment remains a significant hurdle [154, 155].

### Vaccine stability and delivery

The stability and delivery of mRNA vaccines pose significant challenges, particularly in the treatment of GBM. mRNA is inherently unstable and prone to rapid degradation in the body. In addition, the large size of mRNA molecules hinders their uptake by cells. To overcome these challenges, various techniques such as chemical modification and encapsulation using LNPs have been developed to improve both stability and delivery. Innovative formulations such as liposomes, polysomes, and lipoplexes have been explored, with LNPs showing great promise. These LNPs, composed of specific lipid mixtures, are currently being tested in clinical trials to improve the safety and efficacy of mRNA delivery [150, 156, 157].

## Conclusion and future perspectives

mRNA vaccines have shown promise for treating various diseases, especially GBM. They offer advantages such as faster production, affordability, and flexibility, making them a potential option for GBM immunotherapy in the future. However, it is important to acknowledge that the use of mRNA vaccines for GBM treatment is still in the initial stages, and more research and clinical trials are needed to fully understand their therapeutic potential. The efficacy of mRNA vaccines in targeting specific tumor cells and inducing a robust immune response in the GBM tract is an area of ongoing investigation. In the future, advancements in the mRNA vaccine technology and our understanding of the immune response in GBM may lead to the development of more targeted and effective mRNA-based immunotherapies. With continued research and clinical studies, we hope to uncover new strategies to enhance the efficacy of mRNA vaccines and improve outcomes for patients with GBM. Overall, while mRNA vaccines hold promise for the future of GBM treatment, further research is required to validate their effectiveness and optimize their therapeutic role in combating this challenging disease.

## Data Availability

No datasets were generated or analysed during the current study.

## Abbreviations

APCs: Antigen-presenting cells
BBB: Blood–brain barrier
BTSCs: Brain tumor stem cells
CAR: Chimeric antigen receptor
CCL3: C-C motif chemokine ligand 3
CMV: Cytomegalovirus
CNE: Cationic nanoemulsion
CNS: Central nervous system
COVID-19: Coronavirus Disease 2019
CSCs: Cancer stem cells
CT: Computed tomography
DCs: Dendritic cells
DI-TMZ: Dose-intensified temozolomide
DLT: Dose-limiting toxicity
DOTAP/DOPE: 1,2-Dioleoyl-3trimethylammonium-propane/1,2-dioleoyl-sn-glycero-3-phosphoethanolamine
dsRNA: Double-stranded RNA
DTI: Diffusion tensor imaging
eIF4E: Eukaryotic translation initiation factor 4E
EMA: European Medicines Agency
GBMs: Glioblastomas
GEPIA: Gene expression profiling interactive analysis
GM-CSF: Granulocyte-macrophage colony-stimulating factor
GSCs: GBM stem cells
IFN: Interferon
ISGs: IFN-stimulated genes
IVT: In vitro transcription
KPS: Karnofsky performance status
LNPs: Lipid nanoparticles
m6A: *N*6-Methyladenosine
MDSCs: Myeloid-derived suppressor cells
MRI: Magnetic resonance imaging
MTD: Maximum tolerated dose
NCI: National Cancer Institute
NSPCs: Neural stem and progenitor cells
OAS: Oligoadenylate synthetase
ORF: Open reading frame
OS: Overall survival
PAMPs: Pathogen-associated molecular patterns
PFS: Prolonged progression-free survival
PKR: Protein kinase R
PRRs: Pattern recognition receptors
rNTPs: Ribonucleoside triphosphates
ssRNA: Single-stranded RNA
TAAs: Tumor-associated antigens
TAMCs: Tumor-associated myeloid cells
TAMs: Tumor-associated macrophages
TCRs: T cell receptors
Td: Tetanus/diphtheria
TGF-β: Transforming growth factor-Β
Th1: T-help 1 cell
TLRs: Toll-like receptors
TME: Tumor microenvironment
TMZ: Temozolomide
T_Regs_: Regulatory T cells
tRNA: Transfer ribonucleic acid
TSCs: Tumor stem cells
VCE: Vaccinia capping enzyme
VDLNs: Vaccine site-draining lymph nodes
WHO: World Health Organization

## References

[1] Wirsching H-G, Weller M. Glioblastoma. In: Malignant brain tumors: state-of-the-art treatment. Cham: Springer; 2017. p. 265–88.

[2] Herholz K, Langen KJ, Schiepers C, Mountz JM. Brain tumors. Semin Nucl Med. 2012;42:356–70. 23026359 10.1053/j.semnuclmed.2012.06.001 PMC3925448

[3] Kleihues P, Louis DN, Scheithauer BW, Rorke LB, Reifenberger G, Burger PC, Cavenee WK. The WHO classification of tumors of the nervous system. J Neuropathol Exp Neurol. 2002;61:215–25 . 11895036 10.1093/jnen/61.3.215

[4] Ohgaki H, Kleihues P. Genetic pathways to primary and secondary glioblastoma. Am J Pathol. 2007;170:1445–53. 17456751 10.2353/ajpath.2007.070011 PMC1854940

[5] Davis ME. Glioblastoma: overview of disease and treatment. Clin J Oncol Nurs. 2016;20:S2-8. 27668386 10.1188/16.CJON.S1.2-8 PMC5123811

[6] Fernandes C, Department of Medical Oncology, Centro Hospitalar de São João, Porto, Portugal, Costa A, Osório L, Lago RC, Linhares P, et al. Current standards of care in glioblastoma therapy. In: Glioblastoma. Codon Publications; 2017. p. 197–241. https://www.ncbi.nlm.nih.gov/books/NBK469987/10.15586/codon.glioblastoma.2017.ch11 29251860

[7] Weiss Lucas C, Faymonville AM, Loução R, Schroeter C, Nettekoven C, Oros-Peusquens A-M, Langen KJ, Shah NJ, Stoffels G, Neuschmelting V, et al. Surgery of motor eloquent glioblastoma guided by TMS-informed tractography: driving resection completeness towards prolonged survival. Front Oncol. 2022;12: 874631. 35692752 10.3389/fonc.2022.874631 PMC9186060

[8] Segura-Collar B, Hiller-Vallina S, de Dios O, Caamaño-Moreno M, Mondejar-Ruescas L, Sepulveda-Sanchez JM, Gargini R. Advanced immunotherapies for glioblastoma: tumor neoantigen vaccines in combination with immunomodulators. Acta Neuropathol Commun. 2023;11:79. 37165457 10.1186/s40478-023-01569-y PMC10171733

[9] Bausart M, Préat V, Malfanti A. Immunotherapy for glioblastoma: the promise of combination strategies. J Exp Clin Cancer Res. 2022;41:35. 35078492 10.1186/s13046-022-02251-2 PMC8787896

[10] Sun Q, Hong Z, Zhang C, Wang L, Han Z, Ma D. Immune checkpoint therapy for solid tumours: clinical dilemmas and future trends. Signal Transduct Target Ther. 2023;8:320. 37635168 10.1038/s41392-023-01522-4 PMC10460796

[11] Sener U, Ruff MW, Campian JL. Immunotherapy in glioblastoma: current approaches and future perspectives. Int J Mol Sci. 2022;23(13):7046. 35806051 10.3390/ijms23137046 PMC9266573

[12] Wu C, Qin C, Long W, Wang X, Xiao K, Liu Q. Tumor antigens and immune subtypes of glioblastoma: the fundamentals of mRNA vaccine and individualized immunotherapy development. J Big Data. 2022;9:92. 35855914 10.1186/s40537-022-00643-x PMC9281265

[13] Yu MW, Quail DF. Immunotherapy for glioblastoma: current progress and challenges. Front Immunol. 2021;12:1637. 10.3389/fimmu.2021.676301 PMC8158294 34054867

[14] Jiang Y, Chen M, Nie H, Yuan Y. PD-1 and PD-L1 in cancer immunotherapy: clinical implications and future considerations. Hum Vaccines Immunother. 2019;15:1111–22. 10.1080/21645515.2019.1571892 PMC6605868 30888929

[15] Liu Y, Chen J, Xu Y, Sun Q. Novel insight into the role of immunotherapy in gastrointestinal cancer. Mol Clin Oncol. 2022;17:1–9. 10.3892/mco.2022.2590 PMC9627118 36338605

[16] Faghfuri E, Pourfarzi F, Faghfouri AH, Abdoli Shadbad M, Hajiasgharzadeh K, Baradaran B. Recent developments of RNA-based vaccines in cancer immunotherapy. Expert Opin Biol Ther. 2021;21:201–18. 32842798 10.1080/14712598.2020.1815704

[17] Eralp Y. Application of mRNA technology in cancer therapeutics. Vaccines. 2022;10:1262. 36016150 10.3390/vaccines10081262 PMC9415393

[18] Van Der Bruggen P, Zhang Y, Chaux P, Stroobant V, Panichelli C, Schultz ES, Chapiro J, Van den Eynde BJ, Brasseur F, Boon T. Tumor-specific shared antigenic peptides recognized by human T cells. Immunol Rev. 2002;188:51–64. 12445281 10.1034/j.1600-065x.2002.18806.x

[19] Anguille S, Smits EL, Lion E, van Tendeloo VF, Berneman ZN. Clinical use of dendritic cells for cancer therapy. Lancet Oncol. 2014;15:e257–67. 24872109 10.1016/S1470-2045(13)70585-0

[20] Vacchelli E, Vitale I, Eggermont A, Fridman WH, Fučíková J, Cremer I, Galon J, Tartour E, Zitvogel L, Kroemer G. Trial watch: dendritic cell-based interventions for cancer therapy. Oncoimmunology. 2013;2: e25771. 24286020 10.4161/onci.25771 PMC3841205

[21] Pilkington EH, Suys EJ, Trevaskis NL, Wheatley AK, Zukancic D, Algarni A, Al-Wassiti H, Davis TP, Pouton CW, Kent SJ. From influenza to COVID-19: lipid nanoparticle mRNA vaccines at the frontiers of infectious diseases. Acta Biomater. 2021;131:16–40. 34153512 10.1016/j.actbio.2021.06.023 PMC8272596

[22] Liu J, Chang J, Jiang Y, Meng X, Sun T, Mao L, Xu Q, Wang M. Fast and efficient CRISPR/Cas9 genome editing in vivo enabled by bioreducible lipid and messenger RNA nanoparticles. Adv Mater. 2019;31: e1902575. 31215123 10.1002/adma.201902575 PMC6732788

[23] Eygeris Y, Patel S, Jozic A, Sahay G. Deconvoluting lipid nanoparticle structure for messenger RNA delivery. Nano Lett. 2020;20:4543–9. 32375002 10.1021/acs.nanolett.0c01386

[24] Diken M, Kreiter S, Selmi A, Britten C, Huber C, Türeci Ö, Sahin U. Selective uptake of naked vaccine RNA by dendritic cells is driven by macropinocytosis and abrogated upon DC maturation. Gene Ther. 2011;18:702–8. 21368901 10.1038/gt.2011.17

[25] Selmi A, Vascotto F, Kautz-Neu K, Türeci Ö, Sahin U, von Stebut E, Diken M, Kreiter S. Uptake of synthetic naked RNA by skin-resident dendritic cells via macropinocytosis allows antigen expression and induction of T-cell responses in mice. Cancer Immunol Immunother. 2016;65:1075–83. 27422115 10.1007/s00262-016-1869-7 PMC11028682

[26] Ringer S. Regarding the action of hydrate of soda, hydrate of ammonia, and hydrate of potash on the ventricle of the frog’s heart. J Physiol. 1882;3:195. 16991317 10.1113/jphysiol.1882.sp000095 PMC1484928

[27] Ja LEE. Sydney Ringer (1834–1910) and Alexis Hartmann (1898–1964). Anaesthesia. 1981;36:1115–21. 7034584 10.1111/j.1365-2044.1981.tb08698.x

[28] Probst J, Weide B, Scheel B, Pichler B, Hoerr I, Rammensee H, Pascolo S. Spontaneous cellular uptake of exogenous messenger RNA in vivo is nucleic acid-specific, saturable and ion dependent. Gene Ther. 2007;14:1175–80. 17476302 10.1038/sj.gt.3302964

[29] Kreiter S, Selmi A, Diken M, Koslowski M, Britten CM, Huber C, Türeci Ö, Sahin U. Intranodal vaccination with naked antigen-encoding RNA elicits potent prophylactic and therapeutic antitumoral immunity. Can Res. 2010;70:9031–40. 10.1158/0008-5472.CAN-10-0699 21045153

[30] Golombek S, Pilz M, Steinle H, Kochba E, Levin Y, Lunter D, Schlensak C, Wendel HP, Avci-Adali M. Intradermal delivery of synthetic mRNA using hollow microneedles for efficient and rapid production of exogenous proteins in skin. Mol Ther-Nucleic Acids. 2018;11:382–92. 29858073 10.1016/j.omtn.2018.03.005 PMC5992458

[31] Kashem SW, Haniffa M, Kaplan DH. Antigen-presenting cells in the skin. Annu Rev Immunol. 2017;35:469–99. 28226228 10.1146/annurev-immunol-051116-052215

[32] Liu T, Liang Y, Huang L. Development and delivery systems of mRNA vaccines. Front Bioeng Biotechnol. 2021;9: 718753. 34386486 10.3389/fbioe.2021.718753 PMC8354200

[33] Zhang N-N, Li X-F, Deng Y-Q, Zhao H, Huang Y-J, Yang G, Huang W-J, Gao P, Zhou C, Zhang R-R. A thermostable mRNA vaccine against COVID-19. Cell. 2020;182:1271-1283.e1216. 32795413 10.1016/j.cell.2020.07.024 PMC7377714

[34] Li X, Qi J, Wang J, Hu W, Zhou W, Wang Y, Li T. Nanoparticle technology for mRNA: delivery strategy, clinical application and developmental landscape. Theranostics. 2024;14:738–60. 38169577 10.7150/thno.84291 PMC10758055

[35] Hussain A, Yang H, Zhang M, Liu Q, Alotaibi G, Irfan M, He H, Chang J, Liang XJ, Weng Y, Huang Y. mRNA vaccines for COVID-19 and diverse diseases. J Control Release. 2022;345:314–33. 35331783 10.1016/j.jconrel.2022.03.032 PMC8935967

[36] Li J, Men K, Gao Y, Wu J, Lei S, Yang Y, Pan H. Single micelle vectors based on lipid/block copolymer compositions as mRNA formulations for efficient cancer immunogene therapy. Mol Pharm. 2021;18:4029–45. 34559545 10.1021/acs.molpharmaceut.1c00461

[37] Hou X, Zaks T, Langer R, Dong Y. Lipid nanoparticles for mRNA delivery. Nat Rev Mater. 2021;6:1078–94. 34394960 10.1038/s41578-021-00358-0 PMC8353930

[38] Buck J, Grossen P, Cullis PR, Huwyler J, Witzigmann D. Lipid-based DNA therapeutics: hallmarks of non-viral gene delivery. ACS Nano. 2019;13:3754–82. 30908008 10.1021/acsnano.8b07858

[39] Malone RW, Felgner PL, Verma IM. Cationic liposome-mediated RNA transfection. Proc Natl Acad Sci. 1989;86:6077–81. 2762315 10.1073/pnas.86.16.6077 PMC297778

[40] Kauffman KJ, Webber MJ, Anderson DG. Materials for non-viral intracellular delivery of messenger RNA therapeutics. J Control Release. 2016;240:227–34. 26718856 10.1016/j.jconrel.2015.12.032

[41] Chen K, Fan N, Huang H, Jiang X, Qin S, Xiao W, Zheng Q, Zhang Y, Duan X, Qin Z. mRNA vaccines against SARS-CoV-2 variants delivered by lipid nanoparticles based on novel ionizable lipids. Adv Func Mater. 2022;32:2204692. 10.1002/adfm.202204692 PMC9349794 35942272

[42] Patel SK, Billingsley MM, Frazee C, Han X, Swingle KL, Qin J, Alameh M-G, Wang K, Weissman D, Mitchell MJ. Hydroxycholesterol substitution in ionizable lipid nanoparticles for mRNA delivery to T cells. J Control Release. 2022;347:521–32. 35569584 10.1016/j.jconrel.2022.05.020 PMC9376797

[43] Steinhagen F, Kinjo T, Bode C, Klinman DM. TLR-based immune adjuvants. Vaccine. 2011;29:3341–55. 20713100 10.1016/j.vaccine.2010.08.002 PMC3000864

[44] Zhang H, You X, Wang X, Cui L, Wang Z, Xu F, Li M, Yang Z, Liu J, Huang P. Delivery of mRNA vaccine with a lipid-like material potentiates antitumor efficacy through Toll-like receptor 4 signaling. Proc Natl Acad Sci. 2021;118: e2005191118. 33547233 10.1073/pnas.2005191118 PMC8017939

[45] Billingsley MM, Hamilton AG, Mai D, Patel SK, Swingle KL, Sheppard NC, June CH, Mitchell MJ. Orthogonal design of experiments for optimization of lipid nanoparticles for mRNA engineering of CAR T cells. Nano Lett. 2021;22:533–42. 34669421 10.1021/acs.nanolett.1c02503 PMC9335860

[46] Li C, Zhou J, Wu Y, Dong Y, Du L, Yang T, Wang Y, Guo S, Zhang M, Hussain A. Core role of hydrophobic core of polymeric nanomicelle in endosomal escape of siRNA. Nano Lett. 2021;21:3680–9. 33596656 10.1021/acs.nanolett.0c04468

[47] Qiu M, Tang Y, Chen J, Muriph R, Ye Z, Huang C, Evans J, Henske EP, Xu Q. Lung-selective mRNA delivery of synthetic lipid nanoparticles for the treatment of pulmonary lymphangioleiomyomatosis. Proc Natl Acad Sci. 2022;119: e2116271119. 35173043 10.1073/pnas.2116271119 PMC8872770

[48] Miao L, Li L, Huang Y, Delcassian D, Chahal J, Han J, Shi Y, Sadtler K, Gao W, Lin J. Delivery of mRNA vaccines with heterocyclic lipids increases anti-tumor efficacy by STING-mediated immune cell activation. Nat Biotechnol. 2019;37:1174–85. 31570898 10.1038/s41587-019-0247-3

[49] Sayour EJ, Grippin A, De Leon G, Stover B, Rahman M, Karachi A, Wummer B, Moore G, Castillo-Caro P, Fredenburg K. Personalized tumor RNA loaded lipid-nanoparticles prime the systemic and intratumoral milieu for response to cancer immunotherapy. Nano Lett. 2018;18:6195–206. 30259750 10.1021/acs.nanolett.8b02179 PMC6597257

[50] Zhou F, Huang L, Li S, Yang W, Chen F, Cai Z, Liu X, Xu W, Lehto V-P, Lächelt U, et al. From structural design to delivery: mRNA therapeutics for cancer immunotherapy. Exploration. 2024;4:20210146. 38855617 10.1002/EXP.20210146 PMC11022630

[51] Grau M, Walker PR, Derouazi M. Mechanistic insights into the efficacy of cell penetrating peptide-based cancer vaccines. Cell Mol Life Sci. 2018;75:2887–96. 29508006 10.1007/s00018-018-2785-0 PMC6061156

[52] Qiu Y, Man RC, Liao Q, Kung KL, Chow MY, Lam JK. Effective mRNA pulmonary delivery by dry powder formulation of PEGylated synthetic KL4 peptide. J Control Release. 2019;314:102–15. 31629037 10.1016/j.jconrel.2019.10.026

[53] Sabari J, Ramirez KA, Schwarzenberger P, Ricciardi T, Macri M, Ryan A, Venhaus R. Abstract B209: phase 1/2 study of mRNA vaccine therapy+ durvalumab (durva)±tremelimumab (treme) in patients with metastatic non-small cell lung cancer (NSCLC). Cancer Immunol Res. 2019;7:B209–B209.

[54] Sköld AE, van Beek JJ, Sittig SP, Bakdash G, Tel J, Schreibelt G, de Vries IJM. Protamine-stabilized RNA as an ex vivo stimulant of primary human dendritic cell subsets. Cancer Immunol Immunother. 2015;64:1461–73. 26275446 10.1007/s00262-015-1746-9 PMC4612318

[55] Scheel B, Teufel R, Probst J, Carralot JP, Geginat J, Radsak M, Jarrossay D, Wagner H, Jung G, Rammensee HG. Toll-like receptor-dependent activation of several human blood cell types by protamine-condensed mRNA. Eur J Immunol. 2005;35:1557–66. 15832293 10.1002/eji.200425656

[56] Fotin-Mleczek M, Duchardt KM, Lorenz C, Pfeiffer R, Ojkic-Zrna S, Probst J, Kallen K-J. Messenger RNA-based vaccines with dual activity induce balanced TLR-7 dependent adaptive immune responses and provide antitumor activity. J Immunother. 2011;34:1–15. 21150709 10.1097/CJI.0b013e3181f7dbe8

[57] Scheel B, Aulwurm S, Probst J, Stitz L, Hoerr I, Rammensee HG, Weller M, Pascolo S. Therapeutic anti-tumor immunity triggered by injections of immunostimulating single-stranded RNA. Eur J Immunol. 2006;36:2807–16. 17013976 10.1002/eji.200635910

[58] Scheel B, Braedel S, Probst J, Carralot JP, Wagner H, Schild H, Jung G, Rammensee HG, Pascolo S. Immunostimulating capacities of stabilized RNA molecules. Eur J Immunol. 2004;34:537–47. 14768059 10.1002/eji.200324198

[59] Kallen K-J, Heidenreich R, Schnee M, Petsch B, Schlake T, Thess A, Baumhof P, Scheel B, Koch SD, Fotin-Mleczek M. A novel, disruptive vaccination technology: self-adjuvanted RNActive® vaccines. Hum Vaccin Immunother. 2013;9:2263–76. 23921513 10.4161/hv.25181 PMC3906413

[60] Schnee M, Vogel AB, Voss D, Petsch B, Baumhof P, Kramps T, Stitz L. An mRNA vaccine encoding rabies virus glycoprotein induces protection against lethal infection in mice and correlates of protection in adult and newborn pigs. PLoS Negl Trop Dis. 2016;10: e0004746. 27336830 10.1371/journal.pntd.0004746 PMC4918980

[61] Jarzebska NT, Mellett M, Frei J, Kündig TM, Pascolo S. Protamine-based strategies for RNA transfection. Pharmaceutics. 2021;13:877. 34198550 10.3390/pharmaceutics13060877 PMC8231816

[62] Ramalho MJ, Andrade S, Loureiro JA, do Carmo Pereira M. Nanotechnology to improve the Alzheimer’s disease therapy with natural compounds. Drug Deliv Transl Res. 2020;10:380–402. 31773421 10.1007/s13346-019-00694-3

[63] Andrade S, Ramalho MJ, Loureiro JA. Polymeric nanoparticles for biomedical applications. Polymers. 2024;16:249. 38257048 10.3390/polym16020249 PMC10821477

[64] Ding L, Li J, Wu C, Yan F, Li X, Zhang S. A self-assembled RNA-triple helix hydrogel drug delivery system targeting triple-negative breast cancer. J Mater Chem B. 2020;8:3527–33. 31737891 10.1039/c9tb01610d

[65] Melnick K, Dastmalchi F, Mitchell D, Rahman M, Sayour E. Contemporary RNA therapeutics for glioblastoma. NeuroMol Med. 2022;24:8–12. 10.1007/s12017-021-08669-9 PMC8186014 34101090

[66] Youn H, Chung J-K. Modified mRNA as an alternative to plasmid DNA (pDNA) for transcript replacement and vaccination therapy. Expert Opin Biol Ther. 2015;15:1337–48. 26125492 10.1517/14712598.2015.1057563 PMC4696419

[67] Mockey M, Gonçalves C, Dupuy FP, Lemoine FM, Pichon C, Midoux P. mRNA transfection of dendritic cells: synergistic effect of ARCA mRNA capping with Poly (A) chains in cis and in trans for a high protein expression level. Biochem Biophys Res Commun. 2006;340:1062–8. 16403444 10.1016/j.bbrc.2005.12.105

[68] Van Tendeloo VF, Ponsaerts P, Berneman ZN. mRNA-based gene transfer as a tool for gene and cell therapy. Curr Opin Mol Ther. 2007;9:423–31. 17932806

[69] Benteyn D, Anguille S, Van Lint S, Heirman C, Van Nuffel AM, Corthals J, Ochsenreither S, Waelput W, Van Beneden K, Breckpot K. Design of an optimized Wilms’ tumor 1 (WT1) mRNA construct for enhanced WT1 expression and improved immunogenicity in vitro and in vivo. Mol Ther-Nucleic Acids. 2013;2: e134. 24253259 10.1038/mtna.2013.54 PMC3889186

[70] Petsch B, Schnee M, Vogel AB, Lange E, Hoffmann B, Voss D, Schlake T, Thess A, Kallen K-J, Stitz L. Protective efficacy of in vitro synthesized, specific mRNA vaccines against influenza A virus infection. Nat Biotechnol. 2012;30:1210–6. 23159882 10.1038/nbt.2436

[71] Kariko K, Weissman D. Naturally occurring nucleoside modifications suppress the immunostimulatory activity of RNA: implication for therapeutic RNA development. Curr Opin Drug Discov Dev. 2007;10:523. 17786850

[72] Anderson BR, Muramatsu H, Nallagatla SR, Bevilacqua PC, Sansing LH, Weissman D, Karikó K. Incorporation of pseudouridine into mRNA enhances translation by diminishing PKR activation. Nucleic Acids Res. 2010;38:5884–92. 20457754 10.1093/nar/gkq347 PMC2943593

[73] Karikó K, Buckstein M, Ni H, Weissman D. Suppression of RNA recognition by Toll-like receptors: the impact of nucleoside modification and the evolutionary origin of RNA. Immunity. 2005;23:165–75. 16111635 10.1016/j.immuni.2005.06.008

[74] Karikó K, Muramatsu H, Welsh FA, Ludwig J, Kato H, Akira S, Weissman D. Incorporation of pseudouridine into mRNA yields superior nonimmunogenic vector with increased translational capacity and biological stability. Mol Ther. 2008;16:1833–40. 18797453 10.1038/mt.2008.200 PMC2775451

[75] Kormann MS, Hasenpusch G, Aneja MK, Nica G, Flemmer AW, Herber-Jonat S, Huppmann M, Mays LE, Illenyi M, Schams A. Expression of therapeutic proteins after delivery of chemically modified mRNA in mice. Nat Biotechnol. 2011;29:154–7. 21217696 10.1038/nbt.1733

[76] Hacein-Bey-Abina S, Garrigue A, Wang GP, Soulier J, Lim A, Morillon E, Clappier E, Caccavelli L, Delabesse E, Beldjord K. Insertional oncogenesis in 4 patients after retrovirus-mediated gene therapy of SCID-X1. J Clin Investig. 2008;118:3132–42. 18688285 10.1172/JCI35700 PMC2496963

[77] Hacein-Bey-Abina S, Hauer J, Lim A, Picard C, Wang GP, Berry CC, Martinache C, Rieux-Laucat F, Latour S, Belohradsky BH. Efficacy of gene therapy for X-linked severe combined immunodeficiency. N Engl J Med. 2010;363:355–64. 20660403 10.1056/NEJMoa1000164 PMC2957288

[78] Liu Q, Wang X, Liu X, Kumar S, Gochman G, Ji Y, Liao Y-P, Chang CH, Situ W, Lu J. Use of polymeric nanoparticle platform targeting the liver to induce treg-mediated antigen-specific immune tolerance in a pulmonary allergen sensitization model. ACS Nano. 2019;13:4778–94. 30964276 10.1021/acsnano.9b01444 PMC6506187

[79] Thorp EB, Boada C, Jarbath C, Luo X. Nanoparticle platforms for antigen-specific immune tolerance. Front Immunol. 2020;11:945. 32508829 10.3389/fimmu.2020.00945 PMC7251028

[80] Yim EY, Zhou AC, Yim YC, Wang X, Xia T. Antigen-specific mRNA lipid nanoparticle platforms for the prevention and treatment of allergy and autoimmune diseases. BMEMat. 2024;2: e12060.

[81] Kim J-H, Kim BS, Lee S-K. Regulatory T cells in tumor microenvironment and approach for anticancer immunotherapy. Immune Netw. 2020;20: e4. 32158592 10.4110/in.2020.20.e4 PMC7049587

[82] Lorentzen CL, Haanen JB, Met Ö, Svane IM. Clinical advances and ongoing trials on mRNA vaccines for cancer treatment. Lancet Oncol. 2022;23:e450–8. 36174631 10.1016/S1470-2045(22)00372-2 PMC9512276

[83] Nguyen KG, Vrabel MR, Mantooth SM, Hopkins JJ, Wagner ES, Gabaldon TA, Zaharoff DA. Localized interleukin-12 for cancer immunotherapy. Front Immunol. 2020;11: 575597. 33178203 10.3389/fimmu.2020.575597 PMC7593768

[84] Ramanathan A, Robb GB, Chan S-H. mRNA capping: biological functions and applications. Nucleic Acids Res. 2016;44:7511–26. 27317694 10.1093/nar/gkw551 PMC5027499

[85] Yisraeli JK, Melton DA. [4] Synthesis of long, capped transcripts in vitro by SP6 and T7 RNA polymerases. In: Methods in enzymology, vol. 180. Cambridge: Elsevier; 1989. p. 42–50. 10.1016/0076-6879(89)80090-4 2559301

[86] Sweeney R, Fan Q, Yao M-C. Antisense ribosomes: rRNA as a vehicle for antisense RNAs. Proc Natl Acad Sci. 1996;93:8518–23. 8710902 10.1073/pnas.93.16.8518 PMC38704

[87] Timchenko LT. Myotonic dystrophy: the role of RNA CUG triplet repeats. Am J Hum Genet. 1999;64:360–4. 9973273 10.1086/302268 PMC1377745

[88] Al-Saif M, Khabar KS. UU/UA dinucleotide frequency reduction in coding regions results in increased mRNA stability and protein expression. Mol Ther. 2012;20:954–9. 22434136 10.1038/mt.2012.29 PMC3345983

[89] Rabinovich PM, Komarovskaya ME, Ye Z-J, Imai C, Campana D, Bahceci E, Weissman SM. Synthetic messenger RNA as a tool for gene therapy. Hum Gene Ther. 2006;17:1027–35. 17007566 10.1089/hum.2006.17.1027

[90] Tcherepanova IY, Adams MD, Feng X, Hinohara A, Horvatinovich J, Calderhead D, Healey D, Nicolette CA. Ectopic expression of a truncated CD40L protein from synthetic post-transcriptionally capped RNA in dendritic cells induces high levels of IL-12 secretion. BMC Mol Biol. 2008;9:1–13. 18928538 10.1186/1471-2199-9-90 PMC2576345

[91] Seclì L, Leoni G, Ruzza V, Siani L, Cotugno G, Scarselli E, D'Alise AM. Personalized Cancer Vaccines Go Viral: Viral Vectors in the Era of Personalized Immunotherapy of Cancer. Int J Mol Sci. 2023;24(23):16591. 10.3390/ijms242316591. PMID: 38068911; PMCID: PMC10706435. 10.3390/ijms242316591 PMC10706435 38068911

[92] Wang B, Pei J, Xu S, Liu J, Yu J. Recent advances in mRNA cancer vaccines: meeting challenges and embracing opportunities. Front Immunol. 2023;14:1246682. 37744371 10.3389/fimmu.2023.1246682 PMC10511650

[93] Saxena M, van der Burg SH, Melief CJ, Bhardwaj N. Therapeutic cancer vaccines. Nat Rev Cancer. 2021;21:360–78. 33907315 10.1038/s41568-021-00346-0

[94] Smith CC, Selitsky SR, Chai S, Armistead PM, Vincent BG, Serody JS. Alternative tumour-specific antigens. Nat Rev Cancer. 2019;19:465–78. 31278396 10.1038/s41568-019-0162-4 PMC6874891

[95] Schumacher TN, Scheper W, Kvistborg P. Cancer neoantigens. Annu Rev Immunol. 2019;37:173–200. 30550719 10.1146/annurev-immunol-042617-053402

[96] Apavaloaei A, Hardy M-P, Thibault P, Perreault C. The origin and immune recognition of tumor-specific antigens. Cancers. 2020;12:2607. 32932620 10.3390/cancers12092607 PMC7565792

[97] Yarchoan M, Johnson BA, Lutz ER, Laheru DA, Jaffee EM. Targeting neoantigens to augment antitumour immunity. Nat Rev Cancer. 2017;17:209–22. 28233802 10.1038/nrc.2016.154 PMC5575801

[98] Pedersen SR, Sørensen MR, Buus S, Christensen JP, Thomsen AR. Comparison of vaccine-induced effector CD8 T cell responses directed against self-and non-self-tumor antigens: implications for cancer immunotherapy. J Immunol. 2013;191:3955–67. 24018273 10.4049/jimmunol.1300555

[99] Boussiotis VA, Charest A. Immunotherapies for malignant glioma. Oncogene. 2018;37:1121–41. 29242608 10.1038/s41388-017-0024-z PMC5828703

[100] Liu F, Hon GC, Villa GR, Turner KM, Ikegami S, Yang H, Ye Z, Li B, Kuan S, Lee AY, et al. EGFR mutation promotes glioblastoma through epigenome and transcription factor network remodeling. Mol Cell. 2015;60:307–18. 26455392 10.1016/j.molcel.2015.09.002 PMC4609298

[101] Chen DS, Mellman I. Elements of cancer immunity and the cancer–immune set point. Nature. 2017;541:321–30. 28102259 10.1038/nature21349

[102] Zhang Z, Lu M, Qin Y, Gao W, Tao L, Su W, Zhong J. Neoantigen: a new breakthrough in tumor immunotherapy. Front Immunol. 2021;12: 672356. 33936118 10.3389/fimmu.2021.672356 PMC8085349

[103] Hackl H, Charoentong P, Finotello F, Trajanoski Z. Computational genomics tools for dissecting tumour–immune cell interactions. Nat Rev Genet. 2016;17:441–58. 27376489 10.1038/nrg.2016.67

[104] Zhu G, Zhang Q, Zhang J, Liu F. Targeting tumor-associated antigen: a promising CAR-T therapeutic strategy for glioblastoma treatment. Front Pharmacol. 2021;12: 661606. 34248623 10.3389/fphar.2021.661606 PMC8264285

[105] Lin H, Wang K, Xiong Y, Zhou L, Yang Y, Chen S, Xu P, Zhou Y, Mao R, Lv G, et al. Identification of tumor antigens and immune subtypes of glioblastoma for mRNA vaccine development. Front Immunol. 2022;13: 773264. 35185876 10.3389/fimmu.2022.773264 PMC8847306

[106] Ma S, Ba Y, Ji H, Wang F, Du J, Hu S. Recognition of tumor-associated antigens and immune subtypes in glioma for mRNA vaccine development. Front Immunol. 2021;12: 738435. 34603319 10.3389/fimmu.2021.738435 PMC8484904

[107] Ye L, Wang L, Yang J, Hu P, Zhang C, Tong S, Liu Z, Tian D. Identification of tumor antigens and immune landscape in glioblastoma for mRNA vaccine development. Front Genet. 2021;12: 701065. 34527020 10.3389/fgene.2021.701065 PMC8435740

[108] Heine A, Juranek S, Brossart P. Clinical and immunological effects of mRNA vaccines in malignant diseases. Mol Cancer. 2021;20:1–20. 33722265 10.1186/s12943-021-01339-1 PMC7957288

[109] Pardi N, Hogan MJ, Porter FW, Weissman D. mRNA vaccines—a new era in vaccinology. Nat Rev Drug Discov. 2018;17:261–79. 29326426 10.1038/nrd.2017.243 PMC5906799

[110] Freund I, Eigenbrod T, Helm M, Dalpke AH. RNA modifications modulate activation of innate toll-like receptors. Genes. 2019;10:92. 30699960 10.3390/genes10020092 PMC6410116

[111] Melamed JR, Hajj KA, Chaudhary N, Strelkova D, Arral ML, Pardi N, Alameh M-G, Miller JB, Farbiak L, Siegwart DJ, et al. Lipid nanoparticle chemistry determines how nucleoside base modifications alter mRNA delivery. J Control Release. 2022;341:206–14. 34801660 10.1016/j.jconrel.2021.11.022 PMC8905090

[112] Granados-Riveron JT, Aquino-Jarquin G. Engineering of the current nucleoside-modified mRNA-LNP vaccines against SARS-CoV-2. Biomed Pharmacother. 2021;142: 111953. 34343897 10.1016/j.biopha.2021.111953 PMC8299225

[113] Poynter SJ, DeWitte-Orr SJ. Understanding viral dsRNA-mediated innate immune responses at the cellular level using a rainbow trout model. Front Immunol. 2018;9:829. 29740439 10.3389/fimmu.2018.00829 PMC5924774

[114] Nellimarla S, Mossman KL. Extracellular dsRNA: its function and mechanism of cellular uptake. J Interferon Cytokine Res. 2014;34:419–26. 24905198 10.1089/jir.2014.0002

[115] Moradian H, Roch T, Anthofer L, Lendlein A, Gossen M. Chemical modification of uridine modulates mRNA-mediated proinflammatory and antiviral response in primary human macrophages. Mol Ther Nucleic Acids. 2022;27:854–69. 35141046 10.1016/j.omtn.2022.01.004 PMC8807976

[116] Baiersdörfer M, Boros G, Muramatsu H, Mahiny A, Vlatkovic I, Sahin U, Karikó K. A facile method for the removal of dsRNA contaminant from in vitro-transcribed mRNA. Mol Ther Nucleic Acids. 2019;15:26–35. 30933724 10.1016/j.omtn.2019.02.018 PMC6444222

[117] Xu S, Yang K, Li R, Zhang L. mRNA vaccine era—mechanisms, drug platform and clinical prospection. Int J Mol Sci. 2020;21:6582. 32916818 10.3390/ijms21186582 PMC7554980

[118] Seneff S, Nigh G, Kyriakopoulos AM, McCullough PA. Innate immune suppression by SARS-CoV-2 mRNA vaccinations: the role of G-quadruplexes, exosomes, and microRNAs. Food Chem Toxicol. 2022;164: 113008. 35436552 10.1016/j.fct.2022.113008 PMC9012513

[119] Furuichi Y. Discovery of m(7)G-cap in eukaryotic mRNAs. Proc Jpn Acad Ser B Phys Biol Sci. 2015;91:394–409. 26460318 10.2183/pjab.91.394 PMC4729855

[120] Decroly E, Ferron F, Lescar J, Canard B. Conventional and unconventional mechanisms for capping viral mRNA. Nat Rev Microbiol. 2012;10:51–65. 10.1038/nrmicro2675 PMC7097100 22138959

[121] Muttach F, Muthmann N, Rentmeister A. Synthetic mRNA capping. Beilstein J Org Chem. 2017;13:2819–32. 30018667 10.3762/bjoc.13.274 PMC5753152

[122] Galloway A, Cowling VH. mRNA cap regulation in mammalian cell function and fate. Biochim Biophys Acta Gene Regul Mech. 2019;1862:270–9. 30312682 10.1016/j.bbagrm.2018.09.011 PMC6414751

[123] Fuchs AL, Neu A, Sprangers R. A general method for rapid and cost-efficient large-scale production of 5′ capped RNA. RNA. 2016;22:1454–66. 27368341 10.1261/rna.056614.116 PMC4986899

[124] Kyrieleis OJ, Chang J, de la Peña M, Shuman S, Cusack S. Crystal structure of vaccinia virus mRNA capping enzyme provides insights into the mechanism and evolution of the capping apparatus. Structure. 2014;22:452–65. 24607143 10.1016/j.str.2013.12.014 PMC4010090

[125] Parvathy ST, Udayasuriyan V, Bhadana V. Codon usage bias. Mol Biol Rep. 2022;49:539–65. 34822069 10.1007/s11033-021-06749-4 PMC8613526

[126] Chan CY, Carmack CS, Long DD, Maliyekkel A, Shao Y, Roninson IB, Ding Y. A structural interpretation of the effect of GC-content on efficiency of RNA interference. BMC Bioinform. 2009;10:S33. 10.1186/1471-2105-10-S1-S33 PMC2648742 19208134

[127] Mauro VP, Chappell SA. A critical analysis of codon optimization in human therapeutics. Trends Mol Med. 2014;20:604–13. 25263172 10.1016/j.molmed.2014.09.003 PMC4253638

[128] Wang Y, Zhang Z, Luo J, Han X, Wei Y, Wei X. mRNA vaccine: a potential therapeutic strategy. Mol Cancer. 2021;20:33. 33593376 10.1186/s12943-021-01311-z PMC7884263

[129] McNamara MA, Nair SK, Holl EK. RNA-based vaccines in cancer immunotherapy. J Immunol Res. 2015;2015: 794528. 26665011 10.1155/2015/794528 PMC4668311

[130] Banzhoff A, Pellegrini M, Del Giudice G, Fragapane E, Groth N, Podda A. MF59-adjuvanted vaccines for seasonal and pandemic influenza prophylaxis. Influenza Other Respir Viruses. 2008;2:243–9. 19453401 10.1111/j.1750-2659.2008.00059.x PMC4634121

[131] Brito LA, Chan M, Shaw CA, Hekele A, Carsillo T, Schaefer M, Archer J, Seubert A, Otten GR, Beard CW, et al. A cationic nanoemulsion for the delivery of next-generation RNA vaccines. Mol Ther. 2014;22:2118–29. 25027661 10.1038/mt.2014.133 PMC4429691

[132] Pollard C, Rejman J, De Haes W, Verrier B, Van Gulck E, Naessens T, De Smedt S, Bogaert P, Grooten J, Vanham G, De Koker S. Type I IFN counteracts the induction of antigen-specific immune responses by lipid-based delivery of mRNA vaccines. Mol Ther. 2013;21:251–9. 23011030 10.1038/mt.2012.202 PMC3538310

[133] Vik-Mo EO, Nyakas M, Mikkelsen BV, Moe MC, Due-Tønnesen P, Suso EM, Sæbøe-Larssen S, Sandberg C, Brinchmann JE, Helseth E, et al. Therapeutic vaccination against autologous cancer stem cells with mRNA-transfected dendritic cells in patients with glioblastoma. Cancer Immunol Immunother. 2013;62:1499–509. 23817721 10.1007/s00262-013-1453-3 PMC3755221

[134] Mitchell DA, Batich KA, Gunn MD, Huang MN, Sanchez-Perez L, Nair SK, Congdon KL, Reap EA, Archer GE, Desjardins A, et al. Tetanus toxoid and CCL3 improve dendritic cell vaccines in mice and glioblastoma patients. Nature. 2015;519:366–9. 25762141 10.1038/nature14320 PMC4510871

[135] Zhu P, Li S-Y, Ding J, Fei Z, Sun S-N, Zheng Z-H, Wei D, Jiang J, Miao J-L, Li S-Z, et al. Combination immunotherapy of glioblastoma with dendritic cell cancer vaccines, anti-PD-1 and poly I:C. J Pharm Anal. 2023;13:616–24. 37440907 10.1016/j.jpha.2023.04.012 PMC10334272

[136] Mitchell DA, Cui X, Schmittling RJ, Sanchez-Perez L, Snyder DJ, Congdon KL, Archer GE, Desjardins A, Friedman AH, Friedman HS, et al. Monoclonal antibody blockade of IL-2 receptor α during lymphopenia selectively depletes regulatory T cells in mice and humans. Blood. 2011;118:3003–12. 21768296 10.1182/blood-2011-02-334565 PMC3175779

[137] Batich KA, Reap EA, Archer GE, Sanchez-Perez L, Nair SK, Schmittling RJ, Norberg P, Xie W, Herndon JE 2nd, Healy P, et al. Long-term survival in glioblastoma with cytomegalovirus pp65-targeted vaccination. Clin Cancer Res. 2017;23:1898–909. 28411277 10.1158/1078-0432.CCR-16-2057 PMC5559300

[138] Wang QT, Nie Y, Sun SN, Lin T, Han RJ, Jiang J, Li Z, Li JQ, Xiao YP, Fan YY, et al. Tumor-associated antigen-based personalized dendritic cell vaccine in solid tumor patients. Cancer Immunol Immunother. 2020;69:1375–87. 32078016 10.1007/s00262-020-02496-w PMC11027674

[139] Hogan MJ, Pardi N. mRNA vaccines in the COVID-19 pandemic and beyond. Annu Rev Med. 2022;73:17–39. 34669432 10.1146/annurev-med-042420-112725

[140] Roubidoux EK, Schultz-Cherry S. Animal models utilized for the development of influenza virus vaccines. Vaccines. 2021;9:787. 34358203 10.3390/vaccines9070787 PMC8310120

[141] Knezevic I, Liu MA, Peden K, Zhou T, Kang H-N. Development of mRNA vaccines: scientific and regulatory issues. Vaccines. 2021;9:81. 33498787 10.3390/vaccines9020081 PMC7910833

[142] Eon-Duval A, Burke G. Guidance for industry: guidance for human somatic cell therapy and gene therapy guidance for industry: guidance for human somatic cell therapy and gene therapy, 1998. J Chromatogr B Anal Technol Biomed Life Sci. 2004;804:327–35. 10.1016/j.jchromb.2004.01.033 15081927

[143] Maciulaitis R, D’apote L, Buchanan A, Pioppo L, Schneider CK. Clinical development of advanced therapy medicinal products in Europe: evidence that regulators must be proactive. Mol Ther. 2012;20:479–82. 22378030 10.1038/mt.2012.13 PMC3293601

[144] Hosseinalizadeh H, Rahmati M, Ebrahimi A, O’Connor RS. Current status and challenges of vaccination therapy for glioblastoma. Mol Cancer Ther. 2023;22:435–46. 36779991 10.1158/1535-7163.MCT-22-0503 PMC10155120

[145] Chen Z, Wang X, Yan Z, Zhang M. Identification of tumor antigens and immune subtypes of glioma for mRNA vaccine development. Cancer Med. 2022;11:2711–26. 35285582 10.1002/cam4.4633 PMC9249984

[146] Cruz JVR, Batista C, Afonso BDH, Alexandre-Moreira MS, Dubois LG, Pontes B, Moura Neto V, Mendes FDA. Obstacles to glioblastoma treatment two decades after temozolomide. Cancers. 2022;14:3203. 35804976 10.3390/cancers14133203 PMC9265128

[147] Silver A, Feier D, Ghosh T, Rahman M, Huang J, Sarkisian MR, Deleyrolle LP. Heterogeneity of glioblastoma stem cells in the context of the immune microenvironment and geospatial organization. Front Oncol. 2022;12:1022716. 36338705 10.3389/fonc.2022.1022716 PMC9628999

[148] Lin H, Liu C, Hu A, Zhang D, Yang H, Mao Y. Understanding the immunosuppressive microenvironment of glioma: mechanistic insights and clinical perspectives. J Hematol Oncol. 2024;17:31. 38720342 10.1186/s13045-024-01544-7 PMC11077829

[149] Basheer AS, Abas F, Othman I, Naidu R. Role of inflammatory mediators, macrophages, and neutrophils in glioma maintenance and progression: mechanistic understanding and potential therapeutic applications. Cancers. 2021;13:4226. 34439380 10.3390/cancers13164226 PMC8393628

[150] Weng Y, Li C, Yang T, Hu B, Zhang M, Guo S, Xiao H, Liang X-J, Huang Y. The challenge and prospect of mRNA therapeutics landscape. Biotechnol Adv. 2020;40: 107534. 32088327 10.1016/j.biotechadv.2020.107534

[151] Cuoco JA, Benko MJ, Busch CM, Rogers CM, Prickett JT, Marvin EA. Vaccine-based immunotherapeutics for the treatment of glioblastoma: advances, challenges, and future perspectives. World Neurosurg. 2018;120:302–15. 30196171 10.1016/j.wneu.2018.08.202

[152] Tang X, Peng H, Xu P, Zhang L, Fu R, Tu H, Guo X, Huang K, Lu J, Chen H. Synthetic mRNA-based gene therapy for glioblastoma: TRAIL-mRNA synergistically enhances PTEN-mRNA-based therapy. Mol Ther-Oncolytics. 2022;24:707–18. 35317516 10.1016/j.omto.2022.01.013 PMC8913249

[153] Niazi SK. Non-invasive drug delivery across the blood–brain barrier: a prospective analysis. Pharmaceutics. 2023;15:2599. 38004577 10.3390/pharmaceutics15112599 PMC10674293

[154] Rui Y, Green JJ. Overcoming delivery barriers in immunotherapy for glioblastoma. Drug Deliv Transl Res. 2021;11:2302–16. 34053034 10.1007/s13346-021-01008-2 PMC8164566

[155] Sikpa D, Whittingstall L, Savard M, Lebel R, Côté J, McManus S, Chemtob S, Fortin D, Lepage M, Gobeil F. Pharmacological modulation of blood–brain barrier permeability by kinin analogs in normal and pathologic conditions. Pharmaceuticals. 2020;13:279. 33003415 10.3390/ph13100279 PMC7650794

[156] Deng T, Hasan I, Roy S, Liu Y, Zhang B, Guo B. Advances in mRNA nanomedicines for malignant brain tumor therapy. Smart Mater Med. 2023;4:257–65.

[157] Wadhwa A, Aljabbari A, Lokras A, Foged C, Thakur A. Opportunities and challenges in the delivery of mRNA-based vaccines. Pharmaceutics. 2020;12:102. 32013049 10.3390/pharmaceutics12020102 PMC7076378

[158] Hasan M, Khatun A, Kogure K. Intradermal delivery of naked mRNA vaccines via iontophoresis. Pharmaceutics. 2023;15:2678. 38140019 10.3390/pharmaceutics15122678 PMC10747697

[159] Reichmuth AM, Oberli MA, Jaklenec A, Langer R, Blankschtein D. mRNA vaccine delivery using lipid nanoparticles. Ther Deliv. 2016;7:319–34. 27075952 10.4155/tde-2016-0006 PMC5439223

[160] Buckley M, Araínga M, Maiorino L, Pires IS, Kim BJ, Michaels KK, Dye J, Qureshi K, Zhang Y, Mak H, et al. Visualizing lipid nanoparticle trafficking for mRNA vaccine delivery in non-human primates. bioRxiv. 2024. 10.1101/2024.06.21.600088. 39282391 10.1101/2024.09.05.611456 PMC11398480

[161] Wilson B, Geetha KM. Lipid nanoparticles in the development of mRNA vaccines for COVID-19. J Drug Deliv Sci Technol. 2022;74: 103553. 35783677 10.1016/j.jddst.2022.103553 PMC9238147

[162] Nitika, Wei J, Hui AM. The delivery of mRNA vaccines for therapeutics. Life. 2022;12:1254. 36013433 10.3390/life12081254 PMC9410089

[163] Yousefi Adlsadabad S, Hanrahan JW, Kakkar A. mRNA delivery: challenges and advances through polymeric soft nanoparticles. Int J Mol Sci. 2024;25:1739. 38339015 10.3390/ijms25031739 PMC10855060

[164] Chaudhary N, Weissman D, Whitehead KA. mRNA vaccines for infectious diseases: principles, delivery and clinical translation. Nat Rev Drug Discov. 2021;20:817–38. 34433919 10.1038/s41573-021-00283-5 PMC8386155

[165] Chen W, Zhu Y, He J, Sun X. Path towards mRNA delivery for cancer immunotherapy from bench to bedside. Theranostics. 2024;14:96–115. 38164145 10.7150/thno.89247 PMC10750210

[166] Hoffmann MAG, Yang Z, Huey-Tubman KE, Cohen AA, Gnanapragasam PNP, Nakatomi LM, Storm KN, Moon WJ, Lin PJC, West AP Jr, Bjorkman PJ. ESCRT recruitment to SARS-CoV-2 spike induces virus-like particles that improve mRNA vaccines. Cell. 2023;186:2380-2391.e2389. 37146611 10.1016/j.cell.2023.04.024 PMC10121106

[167] Koppu V, Poloju D, Puvvala B, Madineni K, Balaji S, Sheela CMP, Manchikanti SSC, Moon SM. Current perspectives and future prospects of mRNA vaccines against viral diseases: a brief review. Int J Mol Cell Med. 2022;11:260–72. 37605738 10.22088/IJMCM.BUMS.11.3.260 PMC10440005

[168] Rosa SS, Prazeres DMF, Azevedo AM, Marques MPC. mRNA vaccines manufacturing: challenges and bottlenecks. Vaccine. 2021;39:2190–200. 33771389 10.1016/j.vaccine.2021.03.038 PMC7987532

[169] Buschmann MD, Carrasco MJ, Alishetty S, Paige M, Alameh MG, Weissman D. Nanomaterial delivery systems for mRNA vaccines. Vaccines. 2021;9:65. 33478109 10.3390/vaccines9010065 PMC7836001

[170] Yang L, Gong L, Wang P, Zhao X, Zhao F, Zhang Z, Li Y, Huang W. Recent advances in lipid nanoparticles for delivery of mRNA. Pharmaceutics. 2022;14:2682. 36559175 10.3390/pharmaceutics14122682 PMC9787894

[171] Nakagawa T, Kijima N, Hasegawa K, Ikeda S, Yaga M, Wibowo T, Tachi T, Kuroda H, Hirayama R, Okita Y, et al. Identification of glioblastoma-specific antigens expressed in patient-derived tumor cells as candidate targets for chimeric antigen receptor T cell therapy. Neurooncol Adv. 2023;5: vdac177. 36601313 10.1093/noajnl/vdac177 PMC9798403

[172] Kringel R, Lamszus K, Mohme M. Chimeric antigen receptor T cells in glioblastoma-current concepts and promising future. Cells. 2023;12:1770. 37443804 10.3390/cells12131770 PMC10340625

[173] Land CA, Musich PR, Haydar D, Krenciute G, Xie Q. Chimeric antigen receptor T-cell therapy in glioblastoma: charging the T cells to fight. J Transl Med. 2020;18:428. 33176788 10.1186/s12967-020-02598-0 PMC7659102

[174] Morgan RA, Johnson LA, Davis JL, Zheng Z, Woolard KD, Reap EA, Feldman SA, Chinnasamy N, Kuan CT, Song H, et al. Recognition of glioma stem cells by genetically modified T cells targeting EGFRvIII and development of adoptive cell therapy for glioma. Hum Gene Ther. 2012;23:1043–53. 22780919 10.1089/hum.2012.041 PMC3472555

[175] Miao H, Gale NW, Guo H, Qian J, Petty A, Kaspar J, Murphy AJ, Valenzuela DM, Yancopoulos G, Hambardzumyan D, et al. EphA2 promotes infiltrative invasion of glioma stem cells in vivo through cross-talk with Akt and regulates stem cell properties. Oncogene. 2015;34:558–67. 24488013 10.1038/onc.2013.590 PMC4119862

[176] Fischer U, Struss AK, Hemmer D, Pallasch CP, Steudel WI, Meese E. Glioma-expressed antigen 2 (GLEA2): a novel protein that can elicit immune responses in glioblastoma patients and some controls. Clin Exp Immunol. 2001;126:206–13. 11703362 10.1046/j.1365-2249.2001.01635.x PMC1906187

[177] Zhang S, Zhang C, Song Y, Zhang J, Xu J. Prognostic role of survivin in patients with glioma. Medicine. 2018;97: e0571. 29703049 10.1097/MD.0000000000010571 PMC5944547

[178] Sorokin M, Kholodenko I, Kalinovsky D, Shamanskaya T, Doronin I, Konovalov D, Mironov A, Kuzmin D, Nikitin D, Deyev S, et al. RNA sequencing-based identification of ganglioside GD2-positive cancer phenotype. Biomedicines. 2020;8:142. 32486168 10.3390/biomedicines8060142 PMC7344710

[179] Ozawa T, Brennan CW, Wang L, Squatrito M, Sasayama T, Nakada M, Huse JT, Pedraza A, Utsuki S, Yasui Y, et al. PDGFRA gene rearrangements are frequent genetic events in PDGFRA-amplified glioblastomas. Genes Dev. 2010;24:2205–18. 20889717 10.1101/gad.1972310 PMC2947772

[180] Friese MA, Wischhusen J, Wick W, Weiler M, Eisele G, Steinle A, Weller M. RNA interference targeting transforming growth factor-beta enhances NKG2D-mediated antiglioma immune response, inhibits glioma cell migration and invasiveness, and abrogates tumorigenicity in vivo. Cancer Res. 2004;64:7596–603. 15492287 10.1158/0008-5472.CAN-04-1627

[181] Flüh C, Chitadze G, Adamski V, Hattermann K, Synowitz M, Kabelitz D, Held-Feindt J. NKG2D ligands in glioma stem-like cells: expression in situ and in vitro. Histochem Cell Biol. 2018;149:219–33. 29356965 10.1007/s00418-018-1633-5

